# Disruption of TTDA Results in Complete Nucleotide Excision Repair Deficiency and Embryonic Lethality

**DOI:** 10.1371/journal.pgen.1003431

**Published:** 2013-04-18

**Authors:** Arjan F. Theil, Julie Nonnekens, Barbara Steurer, Pierre-Olivier Mari, Jan de Wit, Charlène Lemaitre, Jurgen A. Marteijn, Anja Raams, Alex Maas, Marcel Vermeij, Jeroen Essers, Jan H. J. Hoeijmakers, Giuseppina Giglia-Mari, Wim Vermeulen

**Affiliations:** 1 Department of Genetics, Erasmus MC, Rotterdam, The Netherlands; 2 CNRS, Institut de Pharmacologie et de Biologie Structurale (IPBS) and Université de Toulouse, UPS, Toulouse, France; 3 Department of Cell Biology, Erasmus MC, Rotterdam, The Netherlands; 4 Department of Pathology, Erasmus MC, Rotterdam, The Netherlands; 5 Department of Vascular Surgery, Erasmus MC, Rotterdam, The Netherlands; 6 Department of Radiation Oncology, Erasmus MC, Rotterdam, The Netherlands; Brandeis University, United States of America

## Abstract

The ten-subunit transcription factor IIH (TFIIH) plays a crucial role in transcription and nucleotide excision repair (NER). Inactivating mutations in the smallest 8-kDa TFB5/TTDA subunit cause the neurodevelopmental progeroid repair syndrome trichothiodystrophy A (TTD-A). Previous studies have shown that TTDA is the only TFIIH subunit that appears not to be essential for NER, transcription, or viability. We studied the consequences of TTDA inactivation by generating a *Ttda* knock-out (*Ttda^−/−^*) mouse-model resembling TTD-A patients. Unexpectedly, *Ttda^−/−^* mice were embryonic lethal. However, in contrast to full disruption of all other TFIIH subunits, viability of *Ttda^−/−^* cells was not affected. Surprisingly, *Ttda^−/−^* cells were completely NER deficient, contrary to the incomplete NER deficiency of TTD-A patient-derived cells. We further showed that TTD-A patient mutations only partially inactivate TTDA function, explaining the relatively mild repair phenotype of TTD-A cells. Moreover, *Ttda^−/−^* cells were also highly sensitive to oxidizing agents. These findings reveal an essential role of TTDA for life, nucleotide excision repair, and oxidative DNA damage repair and identify *Ttda^−/−^* cells as a unique class of TFIIH mutants.

## Author Summary

DNA is under constant attack of various environmental and cellular produced DNA damaging agents. DNA damage hampers normal cell function; however, different DNA repair mechanisms protect our genetic information. Nucleotide Excision Repair is one of the most versatile repair processes, as it removes a large variety of DNA helix-distorting lesions induced by UV light and various chemicals. To remove these lesions, the DNA helix needs to be opened by the transcription/repair factor II H (TFIIH). TFIIH is a multifunctional complex that consists of 10 subunits and plays a fundamental role in opening the DNA helix in both NER and transcription. TTDA, the smallest subunit of TFIIH, was thought to be dispensable for both NER and transcription. However, in this paper, we show for the first time that TTDA is in fact a crucial component of TFIIH for NER. We demonstrate that *Ttda^−/−^* mice are embryonic lethal. We also show that *Ttda^−/−^* mouse cells are the first known viable TFIIH subunit knock-out cells, which are completely NER deficient and sensitive to oxidative agents (showing a new role for TFIIH outside NER and transcription).

## Introduction

DNA-damaging agents are a constant challenge to DNA integrity. A network of DNA-repair systems collectively removes most lesions and safeguards the stability of the genome [1]. Nucleotide excision repair (NER) is one such DNA-repair mechanism capable of removing a wide variety of structurally unrelated DNA helix-distorting lesions, including ultraviolet light (UV)-induced lesions and bulky chemical adducts. Two sub-pathways have been identified: global genome NER (GG-NER), eliminating distorting lesions anywhere in the genome and transcription-coupled NER (TC-NER), focusing only on lesions physically blocking ongoing transcription to permit resumption of gene expression.

DNA repair of helix-distorting lesions requires the helix to be opened at the site of the lesion for efficient incision of the damaged strand [2]. A protein complex essential to this process is basal transcription factor II H (TFIIH). Although TFIIH was initially identified as a general RNA polymerase II transcription initiation factor [3], this multi-subunit complex was subsequently found to have multiple functions: including RNA polymerase I transcription and, activated transcription and cell cycle control [4]–[6]. TFIIH is composed of two sub-complexes: the 7-subunit core complex comprised of xeroderma pigmentosum group B (XPB), xeroderma pigmentosum group D (XPD), p62, p52, p44, p34 and trichothiodystrophy group A (TTDA), and the associated trimeric CDK-activating kinase (CAK) complex involving CDK7, MAT1 and cyclin H.

Mutations in genes encoding for TFIIH subunits (XPB, XPD and TTDA) are associated with a surprisingly heterogeneous range of UV-sensitive clinical syndromes [7], [8], consistent with its diverse cellular functions. These syndromes include the (skin)cancer prone disorder xeroderma pigmentosum (XP); the severe neurodevelopmental and premature-aging conditions Cockayne syndrome (CS) and trichothiodystrophy (TTD) and combined forms of these syndromes, XP-CS [9] and XP-TTD [10].

TTD is a multi-systemic premature-ageing condition, characterized by brittle hair and nails, ichthyosis, and progressive mental and physical retardation [11]. Within the disease subtype known as photosensitive TTD, three TFIIH-coding genes have been found to be mutated: *XPB* [12], *XPD* [13], [14] and *TTDA* [15].

Cells isolated from TTD-A patients present a reduced amount of TFIIH, suggesting that TTDA plays an important role in stabilizing the whole TFIIH complex [15], [16]. *TTDA* encodes for an 8 kDa protein that binds to the TFIIH core components XPD and p52 [17], [18]. Although TTDA appears to be the only core TFIIH subunit that is dispensable for mammalian *in vitro* transcription, its presence stimulates RNA synthesis in a reconstituted transcription assay [19]. Moreover, TTDA was originally identified as a component of the yeast transcription pre-incision complex and appeared to have a role in transcription initiation in the presence of an activator [20]. TTDA resides in two cellular fractions: a TFIIH-bound fraction and a free fraction [21]. During engagement in NER, TTDA binds more tightly to TFIIH and possibly plays a role in stabilizing TFIIH on lesions, thereby facilitating the transition between subsequent NER intermediates [21]. The NER-dependent TFIIH-stabilization role can also partly restore the DNA repair deficiency seen in p52 *D. melanogaster* mutants (Dmp52), when an excess of TTDA molecules are available [19]. TTDA has been thought to be required only to stimulate the helix opening during NER [17]. Up to now, the function of TTDA has been presumed not to be essential for NER but only to make it more efficient [22].

Three mutations within the *TTDA* gene of three non-related patients with TTD have been identified [15]. Patient TTD99RO carries a homozygous transition mutation at codon 56, converting an Arginine to a stop codon, thereby truncating the C-terminal 15 amino acids (i.e. more than 20%) of the protein. Patient TTD1BR is heterozygous for this allele, the other allele encodes for a transition mutation at codon 21 that converts a conserved Leucine to a Proline. Siblings TTD13PV and TTD14PV carry a homozygous mutation in the ATG start codon, aborting TTDA protein synthesis. Intriguingly, despite the rather diverse clearly severe mutations, these patients are surprisingly similar in their expression of the clinical features [15], [23], [24], consistent with the idea that they all represent null-alleles. Yeast strains with a complete *Ttda* deletion are viable [20], whereas complete absence of the TFIIH subunits XPB and XPD is incompatible with life in both mammals and yeast [25]–[27], likely due to their indispensable role in transcription. Together, this suggests that TTDA is not a vital TFIIH component [15].

Primary fibroblasts from the TTD-A patients described above have been extensively examined. Although these studies have provided valuable information on the NER function of TTDA, they do not provide an accurate explanation for all the observed clinical symptoms observed in the patients. This can be explained by the fact that most disease-specific symptoms are not apparent in fibroblasts but in neuronal tissue and epithelial cells (ichthyosis and brittle hair). Existing mouse models with mutations in TFIIH components strikingly mimic the clinical symptoms seen in humans [26]–[28] and have provided important information towards our understanding of the molecular and genetic bases of TFIIH-related diseases. Here we describe the generation and analysis of a *Ttda* knock-out (KO) mouse-model to investigate the molecular mechanism that leads to the TTD-specific phenotype. This investigation has shown in fact that TTDA is an essential protein for repair and embryonic development, arguing previous conclusions that needs to be modified.

## Results

### *Ttda* knock-out mouse model is lethal

**Figure 1:**
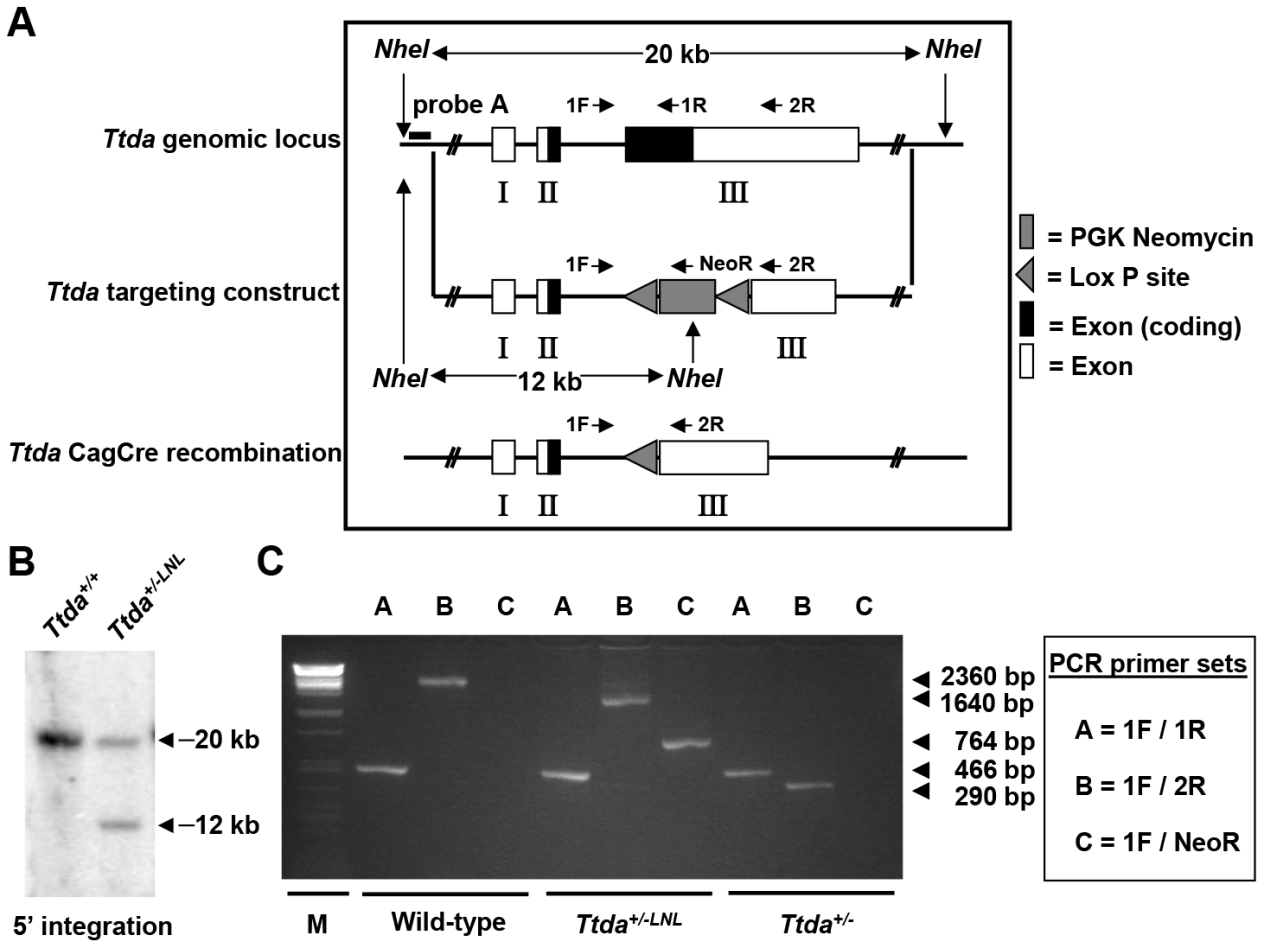
Generation of *Ttda* knock-out mice. (A) Schematic presentation of the mouse *Ttda* genomic locus, *Ttda* targeting construct and *Ttda* locus after CagCre recombination. Roman numbered boxes represent exons: white boxes are non-coding exon (parts) and black boxes are coding exon (parts). In the targeting construct LoxP sites are indicated with gray arrows and the dashed box represents the Neomycin selectable marker driven by a PGK promoter. The positions of NheI restriction sites, size of the NheI restriction fragments and the position of probe A used for DNA blot screening of digested ES cell DNA are indicated. The short arrows indicate the position and direction of primers used for genotyping. (B) DNA blot analysis of NheI-digested ES cell DNA using the probe A. (C) Genotype analysis with diagnostic PCR using primers 1F, 1R, 2R and NeoR in the different combinations (A, B and C).

To study the etiology of TTD and the cell-type-specific consequences of TTDA-deficiency, we developed a *Ttda^−/−^* mouse model. A full KO approach was considered valid, since previous evidence has indicated that TTDA is the sole core TFIIH subunit that is dispensable for viability in yeast [20] and likely also in man [15]. The targeting strategy was designed such that exon 3 (which contains 83% of the *Ttda* coding sequence) was removed and replaced by a neomycin expression cassette, driven by a PGK promoter and flanked by two LoxP sites (referred to as LNL) (Figure 1A).

Following electroporation of the linearized targeting construct into embryonic stem (ES) cells and G418 selection, resistant clones were screened for correct targeting by DNA blotting of NheI-digested genomic DNA with the indicated 5′probe (Figure 1B). We selected two independent ES clones that had undergone homologous recombination and correct 5′ integration and presented a correct karyotype (#J4 and #O8). These clones were injected into C57bl/6 blastocysts to produce chimeric mice with germ-line transmission of the targeted *Ttda* allele (referred to as *Ttda^+/−LNL^*). Male chimeras were mated to C57bl/6 females to produce *Ttda^+/−LNL^* heterozygous offspring. Consistent with the autosomal, recessive nature of the human syndrome the *Ttda^+/−LNL^* mice did not exhibit any obvious phenotype up to the age of 2 years. Both independent mouse lines were used for the generation of *Ttda^−/−^* mice. Since the *Ttda^+/−LNL^* mice still harbored the dominant selectable Neo marker which may interfere with the transcriptional expression of neighboring genes, the *Ttda^+/−LNL^* were crossed with the ubiquitous Cre-recombinase-expressing mouse model [29] to obtain *Ttda^+/−^* offspring. Subsequently, heterozygous mice were inter-crossed to obtain *Ttda^−/−^* mice. Genotyping of offspring was performed by PCR analysis using the sequence-specific primers shown in Figure 1A, and as indicated in Figure 1C. Surprisingly however, *Ttda^−/−^* mice were absent from the large numbers of offspring analyzed (Table 1 and Table S1). It is thus likely that homozygous loss of *Ttda* resulted in embryonic lethality. Additionally, the average litter size observed — when two *Ttda^+/−^* animals were intercrossed — was much lower than the litter size obtained after crossing of *Ttda^+/−^* and wild-type (*Ttda^+/+^*) mice (Table 2), which again points to embryonic loss prior to full gestation. This unexpected lethality seems to contradict with the alleged non-vital function of TTDA in the human situation [15]. Moreover, this lethality is independent of the genetic background of the mouse strain used, since matings of *Ttda^+/−^* neither in C57bl/6 or in FVB background produced viable offspring. Isolated embryos from early pregnancy revealed a normal Mendelian distribution. We observed a progressive loss of phenotypically normal homozygous *Ttda*-deficient embryos during later stages of gestation. *Ttda^−/−^* embryos that do survive up to 19.5 days of gestation show delay in development and have a reduction in size and body-weight (data not shown). Since we were unable to obtain *Ttda^−/−^* mice we attempted to isolate viable *Ttda^−/−^* ES cells and mouse embryo fibroblasts (MEF) lines. We succeeded in establishing both types of cells ruling out that the *Ttda* KO allele causes cellular lethality, as observed with the other TFIIH subunits XPB and XPD due to their indispensable function in basal transcription initiation.

**Table 1:** Genotyping and offspring from *Ttda^+/−^* mice matings.

	Female	Male	Total expected	Total found	Expected % of total	Found % of total
Wild-type	58	60	78	118	25%	35%
*Ttda^+/−^*	90	102	154	192	50%	65%
*Ttda^−/−^*	0	0	78	0	25%	0%
Total	148	162	310	310	100%	100%

Genotyping of offspring from matings of *Ttda^+/−^* mice, distributed over male and females, obtained number and percentage of offspring compared to the theoretical expected figures assuming a Mendalian inheritence pattern.

**Table 2:** Average litter size.

Breeding	Average litter size	SEM	SD
Wild-type×*Ttda^+/−^*	6.3 (n = 17)	0.60	2.40
*Ttda^+/−^*×*Ttda^+/−^*	5.1 (n = 19)	0.36	1.54

Average litter size of matings between two *Ttda^+/−^* animals compared to matings between *Ttda^+/−^* and wild-type mice.

### *Ttda* knock-out cells are NER deficient

**Figure 2:**
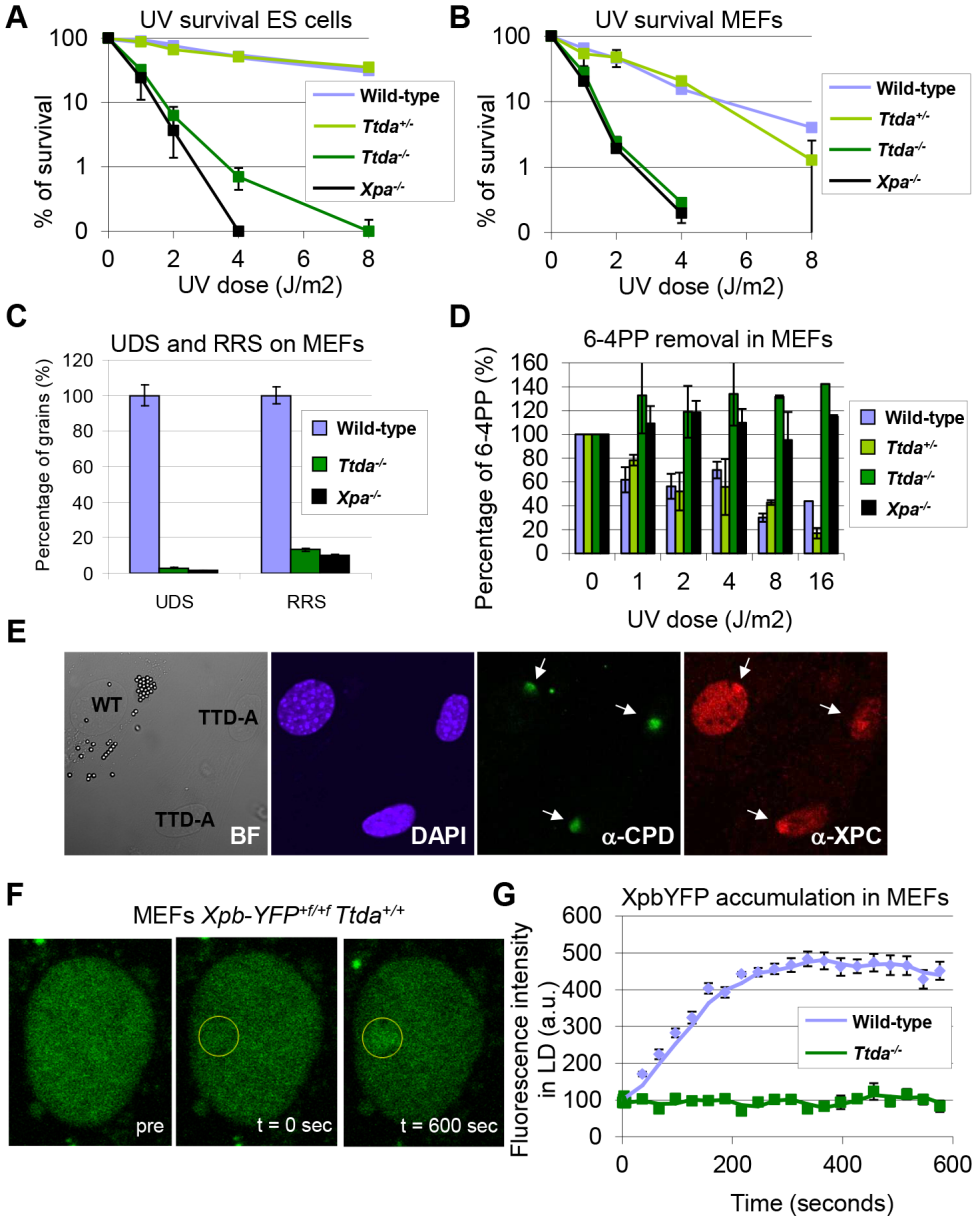
Repair capacity of *Ttda^−/−^* cells. (A) Colony forming ability after different doses of UV of wild-type, *Ttda^+/−^*. *Ttda^−/−^* and *Xpa^−/−^* ES cells. The percentage of surviving cells was plotted against the applied UV-dose, measured by counting surviving colonies of two independent experiments and at least 2 different clones per genotype. The error bars indicate the SEM. (B) Survival assay after different doses of UV of wild-type, *Ttda^+/−^*, *Ttda^−/−^* and *Xpa^−/−^* MEFs. The percentage of surviving cells was plotted against the applied UV-dose, measured by [^3^H]-thymidine incorporation of two independent experiments and at least 2 different clones per genotype. The error bars indicate the SEM. (C) DNA repair synthesis (UDS) and recovery of RNA synthesis (RRS) was measured by autoradiography. For the UDS assay, directly after exposure to 16 J/m^2^ UV-C MEFs were pulse labeled with medium containing [methyl-^3^H] Thymidine, washed with PBS and fixed. For the RRS assay, 16 hours after exposure to 16 J/m^2^ MEFs were pulse labeled with medium containing [^3^H] Uridine, washed with PBS and fixed. UDS and RRS are expressed as the percentage of autoradiographic grains above wild-type, which was set at 100%. (D) 6-4PP removal assayed by ELISA using a 6-4PP specific antibody of wild-type, *Ttda^+/−^*. *Ttda^−/−^* and *Xpa^−/−^* MEFs irradiated with 5 J/m^2^ (UV-light). DNA was isolated at different time-points after UV irradiation (0, 1, 2, 4, 8, and 16 hrs post UV). The amount of 6-4PP measured directly after UV was set at 100%. (E) Immuno-fluorescent analysis of XPC recruitment to local UV-damage in wild-type (labeled with 2 µm latex beads) and *Ttda^−/−^* MEFs. Cells were seeded in a 1∶1 ratio on cover slips and the next day irradiated with 60 J/m^2^ through a filter containing 5 µm pores. Cells were fixed 1 hour after UV and immuno-fluorescent staining was performed using antibodies against CPDs (damage marker, green) and XPC (green). (F) Representative series of confocal images of *XpbYFP^+f/+f^* MEFs before (left; pre), directly after (middle; t = 0 sec) and 600 seconds after (right; t = 600 sec) local UV-damage infliction, the yellow circle marks the area irradiated with the UV-laser. (G) Accumulation kinetics of XpbYFP to local UV-C (laser-induced) DNA damage in a wild-type and *Ttda^−/−^* background. Graphs represents the mean YFP-derived fluorescence intensity at the damaged spot at the indicated time points from approximately 12 cells.

Since homozygous *Ttda^−/−^* mice were not viable, we further analyzed cells derived from *Ttda^−/−^* embryos. We determined the UV sensitivities of ES cells using a clonogenic survival assay. Surprisingly, as shown in Figure 2A, all *Ttda^−/−^* ES clones exhibit a strong UV sensitivity, to the same extent as completely NER-deficient (*Xpa^−/−^*) ES cells [30]. This severe UV-hypersensitivity was unexpected, since we have shown previously that TTD-A human primary fibroblasts only exhibit an intermediate UV sensitivity, caused by slow but persistent repair of UV-induced DNA lesions [22]. To find out whether this remarkable UV sensitivity in *Ttda^−/−^* ES cells is not cell type specific, we also determined the UV sensitivity of *Ttda^−/−^* mouse embryonal fibroblasts (MEFs). As shown in Figure 2B, *Ttda^−/−^* MEFs were also extremely UV-sensitive to the same extent as *Xpa^−/−^* MEFs.

Next, we investigated NER capacity in the *Ttda^−/−^* MEFs, by measuring: UV-induced unscheduled DNA repair synthesis (UDS), i.e. a measure for GG-NER; and recovery of RNA synthesis after UV-irradiation (RRS), i.e. a measure for TC-NER (Figure 2C). UDS and RRS were both severely affected similar to completely NER-deficient (*Xpa^−/−^*) MEFs, in line with the strong UV-hypersensitivity. In addition, *Ttda^−/−^* MEFs were fully deficient in repair of 6-4 pyrimidine-pyrimidone (6-4PP) photoproducts as measured by ELISA, even 16 hours after UV irradiation (Figure 2D). This eliminates the option of slow but persisting repair as observed in the human TTD-A fibroblasts corroborating the complete NER-deficiency in these cells.

### Ttda is required for TFIIH loading in NER complexes

To further dissect the stage at which this unexpected complete NER deficiency occurs in *Ttda^−/−^* MEFs, we first checked whether damage recognition is affected by Ttda ablation. As shown in Figure 2E, the damage recognizing protein Xpc is efficiently loaded to local UV-damage (LUD) similar as in wild-type cells. These results suggests that Ttda functions at the stage of TFIIH-loading onto XPC-bound lesions, downstream of damage recognition. To determine whether the TFIIH complex lacking Ttda was still capable of assembling at UV-induced DNA lesions — as shown in human TTD-A cells [17], [22] — we measured the binding kinetics of TFIIH to damaged regions. In these experiments either a Multi-photon (MP) laser or a UV-laser was used to locally induce (UV) DNA damage in the nuclei of living cells [31]. To monitor TFIIH loading we used a recently developed knock-in mouse model which expresses homozygously a fluorescently-tagged (YFP, for yellow fluorescent protein) Xpb (largest subunit of TFIIH) [32]. We crossed the *Ttda* KO allele into this *XpbYFP^+f/+f^* background and isolated E10.5 MEFs from *XpbYFP^+f/+f^ Ttda^+/−^* matings, each expressing the Xpb-YFP fusion protein. In contrast to the fast accumulation kinetics of Xpb-YFP to UV-laser damaged spots in the wild-type background (Figure 2F and 2G), this protein was unable to accumulate in *Ttda^−/−^* background. Wild-type and *Ttda^+/−^* MEFs showed similar accumulation kinetics for the multi-photon (MP) damaged area, which induces among other lesions also UV photoproducts [31] (Figure S1). However, Xpb-YFP is incapable of accumulating to DNA damaged regions in *Ttda^−/−^* MEFs, even 15 minutes after DNA damage induction.

**Figure 3:**
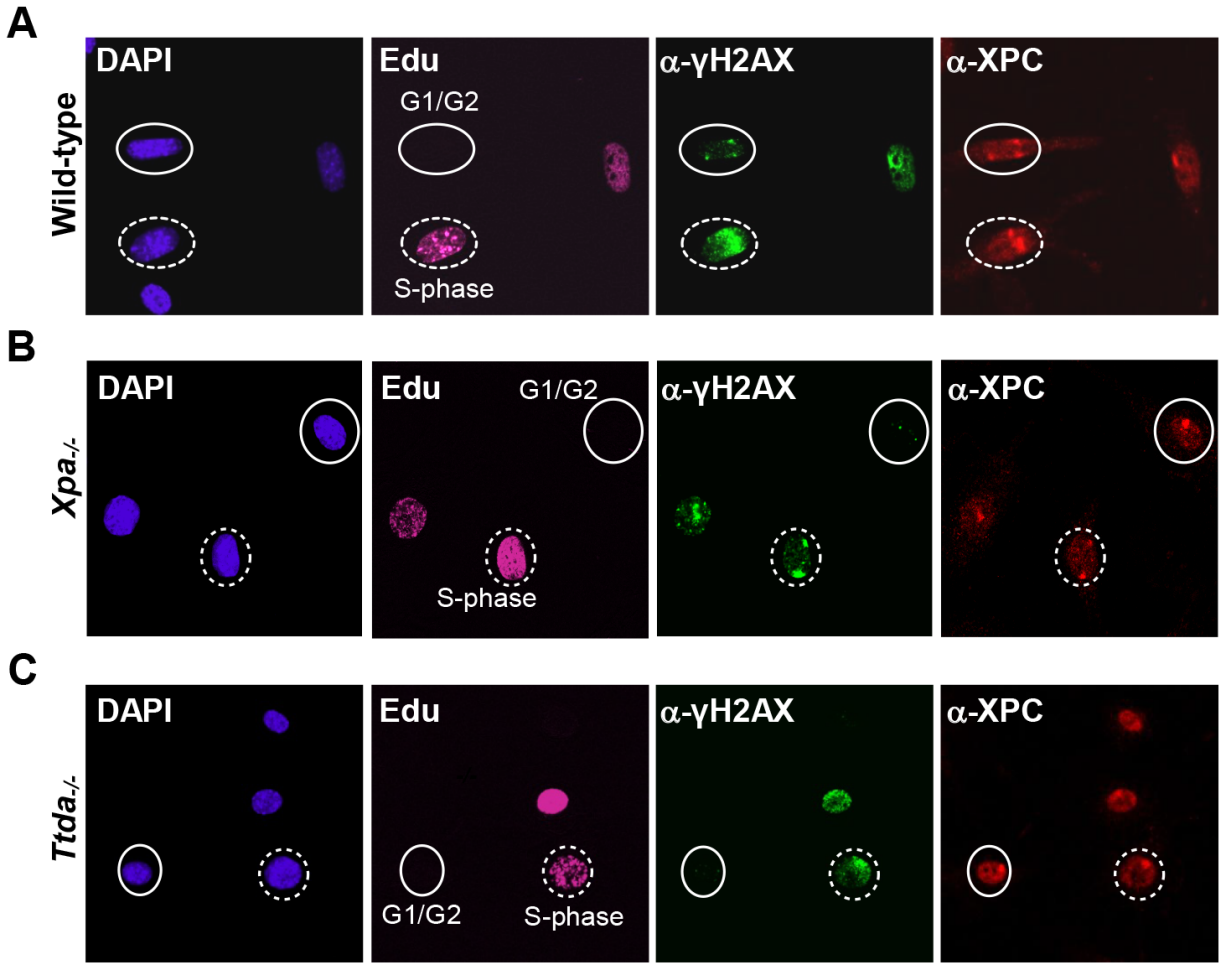
γH2AX signaling is abolished in *Ttda^−/−^* MEFs. Cell cycle dependent analysis of XPC and γH2AX recruitment to local UV-damage in wild-type (A), *Xpa^−/−^* (B) and *Ttda^−/−^* (C) MEFs. Cells were seeded on cover slips and the next day irradiated with 60 J/m^2^ through a filter containing 5 µm pores and subsequently labeled with EdU for 1 hour. After fixation cells were assayed for DNA synthesis using EdU and Alexa Fluor 647 azide (cell cycle marker, pink) and by immuno-fluorescent staining using antibodies against γH2AX (green) and XPC (red). Dashed circles indicate typical examples of cells in S-phase, closed circles indicate typical examples of G1/G2 cells.

The absence of TFIIH binding to Xpc bound DNA lesions suggests that down-stream processing of UV-lesions by the NER machinery is abrogated, since the helicase function of TFIIH is required for further assembly of the pre-incision NER complex and sequential dual incision [2], [33]. Repair intermediates produced by NER incision induces H2AX phosphorylation (γH2AX) in a cell-cycle independent manner [34], [35]. Local γH2AX after filter irradiation can be used as a sensitive marker for dual incision during NER [36]. In non-S-phase cells, since γH2AX signaling in S-phase cells is both triggered by stalled replication forks and NER. A clear local γH2AX signaling is observed in wild-type non-S-phase MEFs 1 hr after LUD, which is however absent in both *Xpa^−/−^* and *Ttda^−/−^* MEFs (Figure 3). Together these data show that Ttda is pivotal for TFIIH loading on UV lesions and that in its absence no NER-dependent dual incision occurs. We therefore consider Ttda as an essential NER factor.

### Knock-down of mutant hTTDA results in complete NER deficiency

**Figure 4:**
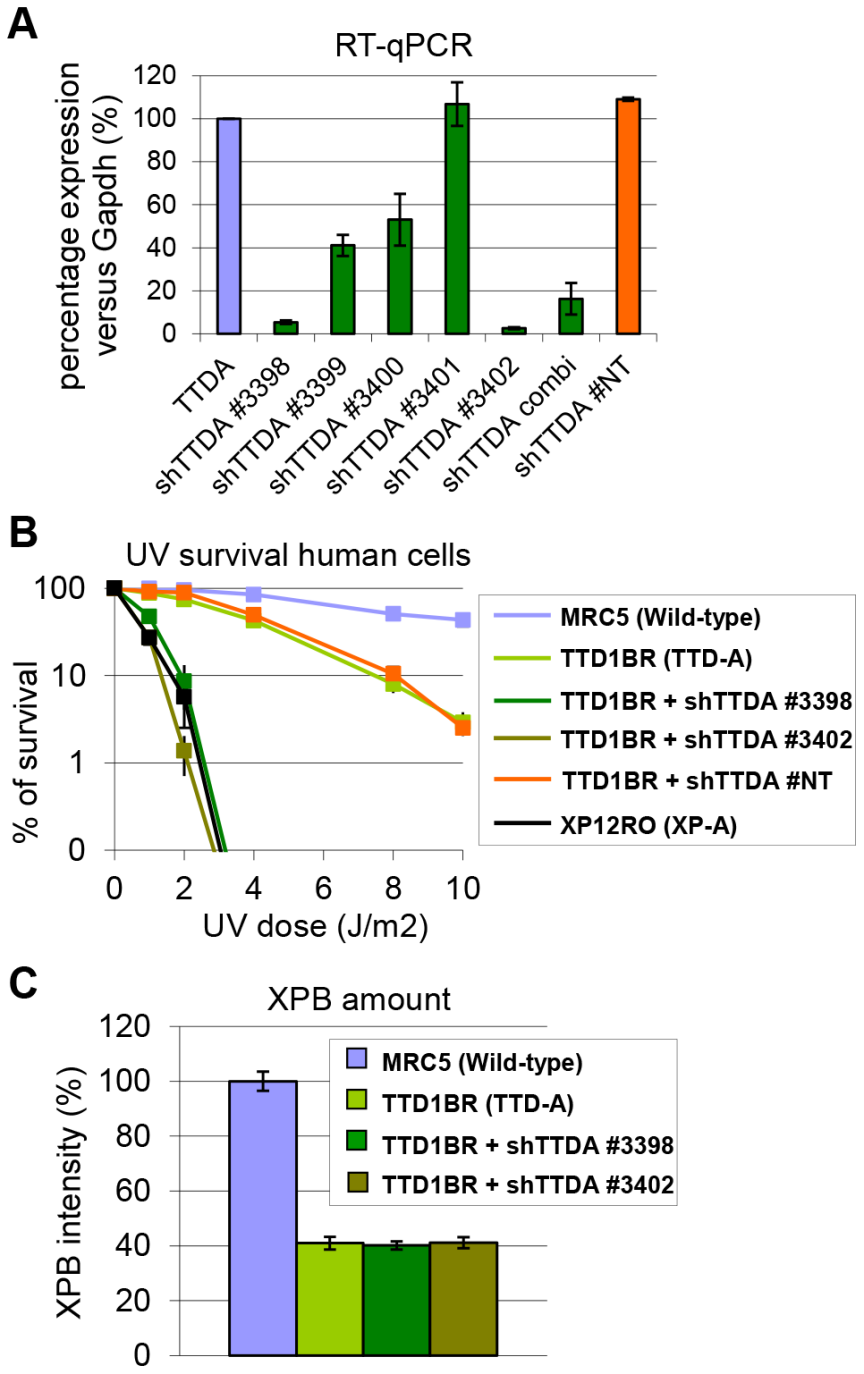
Knock-down of mutant hTTDA results in complete NER deficiency. (A) Relative expression levels of TTDA mRNA as determined by quantitative RT-PCR in TTD1BR-sv cells (TTD-A) and TTD1BR-sv cells stably expressing shRNAs for respectively: #non-targeting (NT), #3398, #3399, #3400, #3401 or #3402. The levels were normalized to *Tubulin* and the error bars indicate SEM between two independent experiments. (B) Colony forming ability after different doses of UV irradiation of MRC5-sv (wild-type), XP12RO-sv (XP-A), TTD1BR-sv and TTD1BR-sv cells stably expressing shRNA: #non-targeting (NT), #3398, #3399, #3400, #3401 and #3402. The percentage of surviving cells was plotted against the applied UV-dose, measured by counting surviving colonies of two independent experiments. The error bars indicate the SEM. (C) Quantitative immuno-fluorescence to determine the relative amount of XPB (TFIIH) in MRC5-sv (wild-type), TTD1BR-sv (TTD-A) and TTD1BR-sv cells stably expressing shRNA (#3398 or #3402). Confocal microscope pictures were used to quantify the average intensity of XBP and MDC1 (internal control) in >100 cells and error bars indicate SEM.

The absence of residual NER activity in *Ttda^−/−^* MEFs and ES cells is in contrast with the partial NER activity in TTD-A human primary fibroblasts [22]. A possible explanation for this apparent discrepancy could be derived from a human-mouse difference in NER efficiency. This could be caused by the virtual absence of CPD removal by GG-NER in rodent cells, which still occurs albeit with a slow rate in human cells [37]. Alternatively, it is not formally excluded that a mutated TTDA protein with partial biological activity is still present in the patient cells. To investigate this latter option we attempted to further reduce TTDA in human patient cells (TTD1BR-SV) by shRNA interference of the resident mutant *TTDA* transcript. The knock-down efficiency of the different shRNAs targeting the *TTDA* transcript was verified by RT-qPCR (Figure 4A). We selected two shRNAs (#3398 and #3402) that were most efficient in reducing the resident *TTDA* transcript to approximately 5% of the initial amount. Next, we determined the UV-sensitivity by performing a clonogenic survival assay (Figure 4B). Surprisingly, depleting mutant *TTDA* mRNA in TTD-A human patient cells severely aggravated the UV-sensitivity, whereas a control non-targeting shRNA did not have any effect. These results suggest that the mutated human TTDA proteins still harbor residual NER activity and that the severe NER-deficient phenotype observed in *Ttda^−/−^* cells is not specific for mouse cells. To find out whether this strong NER deficiency upon TTDA depletion by shRNA in the human cells was due to a further reduction in cellular TFIIH content below a critical threshold, we determined TFIIH levels in the parental TTD-A cells and their cognate shRNA-TTDA-depleted cells. As shown by the immunofluorescence staining of the XPB subunit of TFIIH in Figure 4C and Figure S2 there was no further decline of the already low TFIIH levels upon TTDA depletion by shRNA in TTD-A cells.

### TTDA mutant proteins accumulate at UV-induced damaged regions

**Figure 5:**
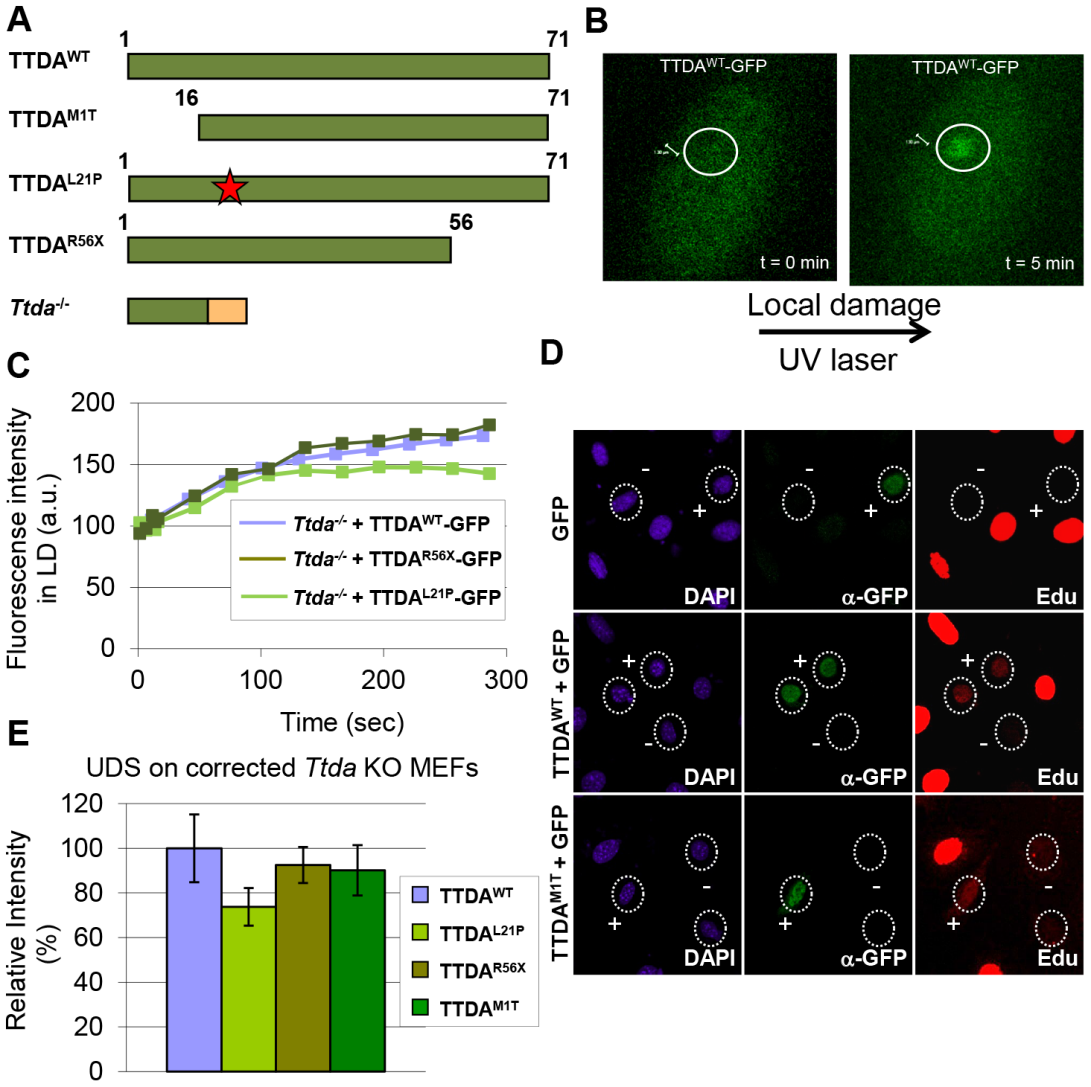
Mutant TTDA protein accumulation at local UV damage. (A) Schematically representation of the predicted TTDA polypeptide length (in amino acids) in human wild-type cells (TTDA^WT^), TTD-A patient cells (TTDA^M1T^, TTDA^L21P^ and TTDA^R56X^) and the *Ttda* knock-out cells (*Ttda^−/−^*). The red star represents the mutation found in TTDA^L21P^ and the red part of the *Ttda^−/−^* bar represents intronic encoded non-sense amino acids. (B) Representative confocal images of *Ttda^−/−^* MEFs expressing TTDA^WT^-GFP before (t = 0 min) and after local UV-damage infliction (t = 5 min) in a selected area inside the nucleus (dashed circle). (C) Accumulation kinetics of TTDA^WT^-GFP, TTDA^L21P^-GFP and TTDA^R56X^-GFP to local UV-C (laser-induced) DNA damage expressed in *Ttda^−/−^* MEFs. Graphs represents the mean GFP-derived fluorescence intensity at the damaged spot at the indicated time points from approximately 12 cells. (D) Representative confocal microscope images of UV-induced UDS of *Ttda^−/−^* MEFs transiently co-transfected with an empty GFP vector (as a marker for transfected cells) in combination with a vector containing TTDA^WT^ or TTDA^M1T^. Cells were seeded on cover slips and transfected 2 days before the experiment. Cells were irradiated with 16 J/m^2^ and subsequently labeled for 2 hours with EdU. Cells were fixed and stained for EdU incorporation (UDS and S-phase DNA synthesis, red) and GFP using antibodies against GFP (transfected cells, green). The intense red labeled cells are cells in S-phase. (E) The percentage of UDS signal in the nucleus was quantified by measuring the average fluorescence intensity from at least 25 cells positively transfected (containing GFP) and non-S-phase cells with TTDA^WT^, TTDA^M1T^, TTDA^R56X^ or TTDA^L21P^. The error bars indicate the SEM.

Since depletion of mutant TTDA aggravated UV-sensitivity, we further investigated the functionality of the mutant human proteins (schematically depicted in Figure 5A). Because TTDA seems to be required for efficient loading of additional NER factors, the ability of TTDA to localize to LUD is indicative of its function in NER. To determine the binding of mutant TTDA to laser-induced LUD in living cells, we transduced *Ttda^−/−^* MEFs with lentiviruses encoding for GFP-tagged TTDA: TTDA^WT^-GFP (wild type), TTDA^M1T^-GFP (start site mutation, using the first downstream Methionine codon (M16)), TTDA^R56X^-GFP (premature stop mutation) or TTDA^L21P^-GFP (transition mutation). The latter three mimic mutations found in TTD-A patients. TTDA-GFP fully complements the UV-sensitivity of Ttda^−/−^ MEFs (Figure S3A and [21]) and shows that the tagged TTDA is biologically active. As shown in Figure 5B and 5C, TTDA^R56X^-GFP and TTDA^L21P^-GFP accumulated with similar initial kinetics as TTDA^WT^-GFP to LUD, though prior to steady-state less TTDA^L21P^-GFP accumulated compared to TTDA^WT^-GFP. This would suggest that the binding time of this mutant protein is reduced compared to the wild-type protein. The ability to accumulate at DNA damaged regions was also confirmed by an immunofluorescence experiment, using a 5 µm filter and a UV-C lamp to apply local UV damage (Figure S3B). However, we did not find accumulation of TTDA^M1T^-GFP, despite the fact that patient cells carrying this mutation display only a mild NER-deficient phenotype [15]. This apparent discrepancy could be explained by a possible combinational functional interference of both the 16 amino acid N-terminal truncation and the C-terminal GFP-tag, despite the notion that over expression of a full length TTDA-GFP rescues the UV-sensitivity of *Ttda^−/−^* MEFs (Figure S3A). To further investigate a possible partial function of the translational start-site mutant, we over expressed non-tagged mutant TTDA cDNA — mimicking the translation start mutation found in TTD13 PV and TTD14 PV patients — and the other mutants in *Ttda^−/−^* MEFs and assayed for UDS. Co-transfected GFP served as a marker to identify transfected cells. Over expression of all TTDA mutant cDNAs clearly corrected the DNA repair synthesis deficiency of *Ttda^−/−^* MEFs to almost wild-type levels, including the translational start-site (M1T) mutant (Figure 5D, 5E). Together these results strongly suggest that TTD-A patients do express a partially functional mutant protein and confirms that also in human cells TTDA is an essential NER component, rather than only an NER accessory factor.

### Lethal phenotype caused by Ttda absence

**Figure 6:**
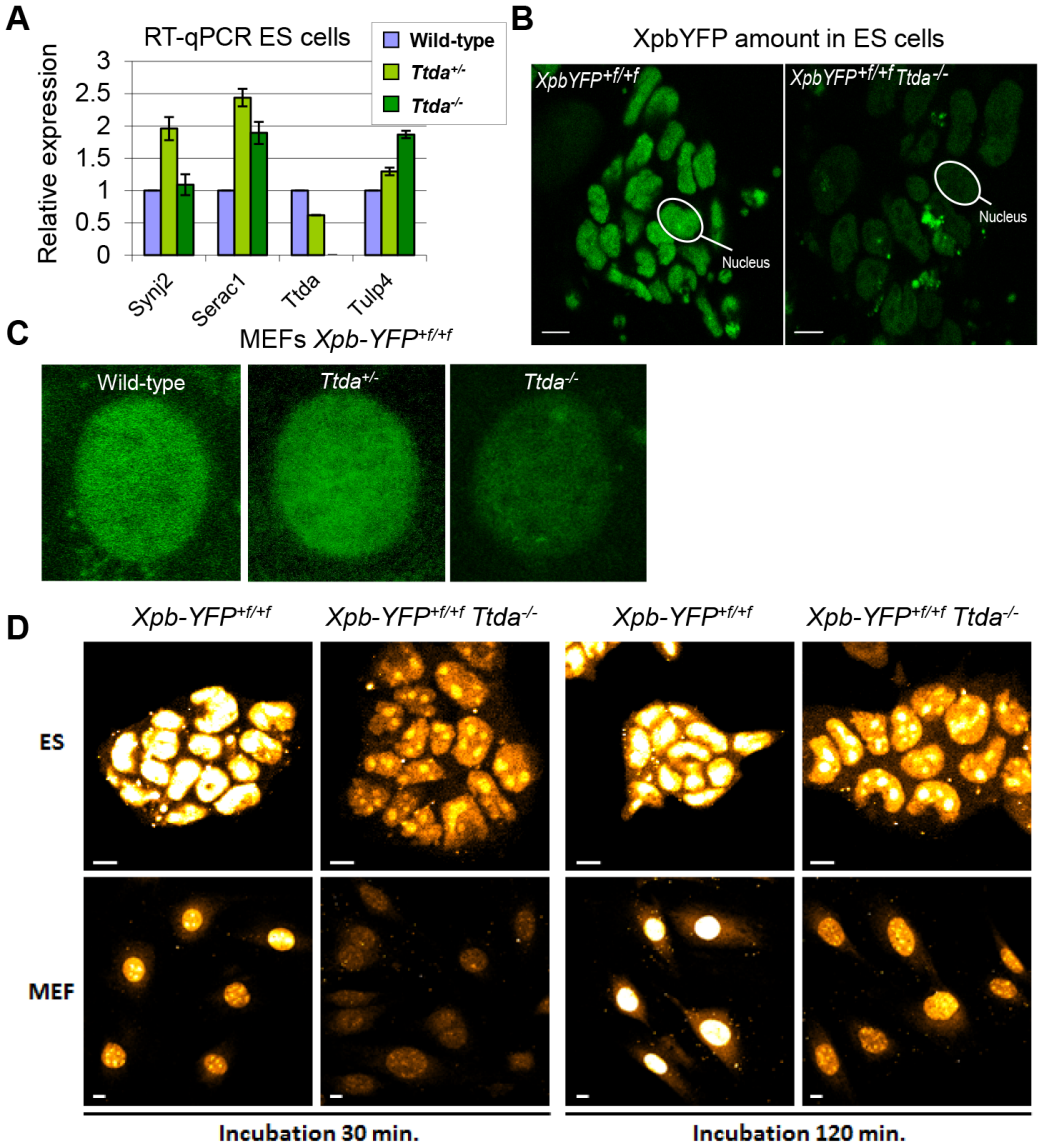
Gene expression levels and TFIIH amount in *Ttda^−/−^* ES cells. (A) Relative expression levels of mRNAs neighboring genes encoding Synaptojamin 2 (*Synj2*), Serine active site containing 1 (*Serac1*), Trichothiodystrophy group A (*Ttda*) and Tubby like protein 4 (*Tulp4*) in *Ttda^−/−^* (n = 2), *Ttda^+/−^* (n = 2) and wild-type (n = 2) ES cells as determined by quantitative RT-PCR. The levels were normalized to *Gapdh* and the error bars indicate SEM between experiments. (B) Representative confocal microscope pictures of *XpbYFP^+f/+f^ Ttda^−/−^*, *XpbYFP^+f/+f^ Ttda^+/−^* and *XpbYFP^+f/+f^* MEFs isolated from 10.5-day-old embryos. (C) Confocal images of ES cells isolated from *XpbYFP^+f/+f^* mouse model (left panel) and from *XpbYFP^+f/+f^ Ttda^−/−^* mouse (right panel). The green signal is the direct fluorescence of the YFP tagged protein. The white bar measures 10 mm. (D) Confocal images of EU incorporation into ES cells isolated from *XpbYFP^+f/+f^* mouse model and from *XpbYFP^+f/+f^ Ttda^−/−^* mouse (upper panel) and MEF's isolated from the same mouse models (lower panel). Two EU incubation times have been performed: 30 minutes (left panels) and 120 minutes (right panels). The white bar measures 10 mm.

The new finding of TTDA's essential role in NER has far-reaching biological significance. However, the complete NER deficiency cannot explain the embryonic lethality, since other mice with fully compromised NER function (such as *Xpa^−/−^* mice) do not display similar developmental abnormalities and are viable [38], [39]. To gain further insight into the nature of this lethal phenotype, we analyzed whether the altered genomic locus of the *Ttda* KO allele interferes with the expression of neighboring genes. The deletion of genomic sequences in the *Ttda* gene may include cryptic or unrecognized transcriptional enhancers or insulators which may create hypomorphic expression of adjacent genes (see [40] for a typical example). To investigate possible transcriptional interference, we analyzed the expression levels of the 3 most proximal neighboring genes to the *Ttda* gene in *Ttda^−/−^* ES cells by quantitative real-time PCR (RT-qPCR): Synaptojanin 2 (*Synj2*, 35.5 Kb upstream), Serine active site containing 1 (*Serac1*, 89 bp upstream) and Tubby-like protein 4 (*Tulp4*, 21.3 Kb downstream). The expression of these genes was not reduced when compared with expression in their heterozygous and wild-type cognates (Figure 6A). Two of these genes showed increased expression in the *Ttda^−/−^* cells. This likely does not cause embryonic lethality, since cells from heterozygous animals, which also had this increased expression do not display any obvious phenotype. Expression analysis of the *Ttda* allele neighboring genes at a later stage of development (E11.5) (Figure S4) confirmed the absence of a clear correlation between aberrant gene expression and lethality in *Ttda^−^* ^/−^ embryos.

It has been suggested that the TTDA protein is a repair-specific TFIIH-subunit, which is not strictly required for basal transcription as the other TFIIH components are [17]. However, mutated TTDA causes an overall reduction of TFIIH protein abundance in human fibroblasts [16]. This sub-limiting amount of TFIIH does not cause a significant reduction of basal transcription within cultured TTD-A patient fibroblasts [16]. On the other hand, in the developing mouse embryo a decreased amount of TFIIH might reduce the transcription capacity which is needed to produce a fully developed animal. To directly monitor the quantity of TFIIH in living cells, we isolated ES cells and E10.5 MEFs from *XpbYFP^+f/+f^ Ttda^+/−^* matings (see above), each expressing the XPB-YFP fusion protein. A clear reduction in the quantity of TFIIH (as deduced from the strong reduction of the YFP signal) was easily observed in the live cell images of both *Ttda* KO ES cells and MEFs (Figure 6B–6C). We determined by direct fluorescence measurements the YFP signal emitted from the nuclei of ES cells and MEFs, which was respectively 22% and 33% in the KO cells as compared to wild-type cells isolated from litter mates. These levels are for the MEFs comparable to the amounts measured in human cells (approximately 30%) [16] and appeared even lower in ES cells.

Next we analyzed the transcription capacity in these murine cells by pulse labeling *de novo* RNA synthesis for either 30 minutes or 2 hours with fluorescent based 5-ethynyl-uridine (EU). The average fluorescence intensity, which is a measure for the total amount of transcription in these cells, was evaluated (Figure 6D). In accordance with the reduced steady-state level of TFIIH, measured in ES cells and MEFs, the overall transcription appeared to be significantly reduced in *Ttda^−/−^* cells as well.

### Ttda knock-out cells are sensitive to oxidative DNA damage

**Figure 7:**
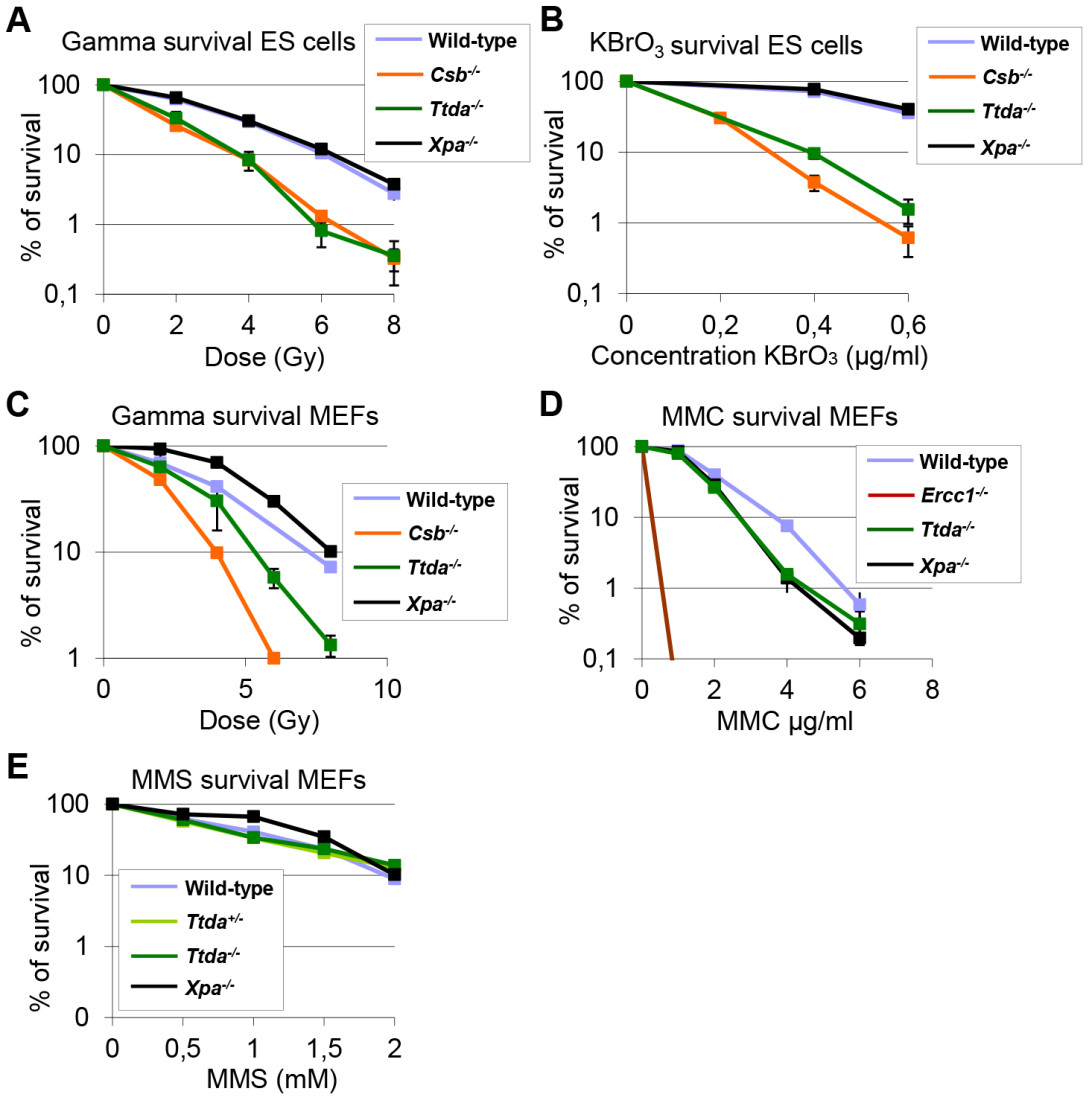
*Ttda^−/−^* ES cells and MEFs exhibit oxidative DNA damage sensitivity. (A) Colony forming ability after different doses of gamma irradiation of wild-type, *Ttda^−/−^*, Csb^−/−^ and *Xpa^−/−^* ES cells. The percentage of surviving cells was plotted against the applied gamma-dose. (B) Colony forming ability after different concentrations of KBrO_3_ of wild-type, *Ttda^−/−^*, *Csb^−/−^* and *Xpa^−/−^* ES cells. The percentage of surviving cells was plotted against the applied gamma-dose. (C) Colony forming ability after different doses of gamma irradiation of wild-type, *Ttda^−/−^*, *Csb^−/−^* and *Xpa^−/−^* MEFs. The percentage of surviving cells was plotted against the applied gamma-dose. (D) Colony forming ability after different doses of 1 hour MMC treatment of wild-type, *Ttda^+/−^*, *Ercc1^−/−^* and *Xpa^−/−^* MEFs. The percentage of surviving cells was plotted against the applied gamma-dose. (E) Colony forming ability after different doses of 1 hour MMS treatment of wild-type, *Ttda^+/−^*, *Ttda^−/−^* and *Xpa^−/−^* MEFs. The percentage of surviving cells was plotted against the applied gamma-dose. For each survival plot (A–E) at least 2 different clones per genotype were measured in two independent experiments. The error bars indicate the SEM.

Despite the severely reduced amount of TFIIH in *Ttda^−/−^* cells, which also attenuated overall transcription, the proliferative capacity of the embryonic cells was not affected (Figure S5A). It is thus not likely that reduced overall transcription would be the sole cause to the observed embryonic lethality. Detailed analysis of different mutant TFIIH mouse models revealed a correlation between sensitivity to oxidative DNA damage and severity of the phenotype of the different models. These observations argued for a unknown function of TFIIH in oxidative lesion removal [27]. To investigate whether *Ttda^−/−^* cells are also defective in repairing other (non NER-type) DNA lesions, we measured their sensitivity to several oxidizing agents. Clonogenic survival assays performed on *Ttda^−/−^* ES cells revealed hyper-sensitivity to gamma irradiation (Figure 7A) and potassium bromate (Figure 7B), similar to *Csb^−/−^* ES cells (known to be sensitive to oxidative DNA damage) [30]. Since the *Xpa^−/−^* ES cells assayed in parallel were not sensitive to any of these agents, this sensitivity is not a general effect of NER-deficiency. This phenomenon is also not cell-type specific, since *Ttda^−/−^* MEFs are also hyper-sensitive to gamma irradiation (Figure 7C). To exclude the possibility that *Ttda^−/−^* cells have a general low tolerance to DNA damage, we also measured their sensitivity to mitomycin C (MMC). MMC induces inter-strand cross-linking that is specifically repaired by the inter-strand cross-link repair pathway — a pathway which specifically involves the NER protein complex XPF-ERCC1 (other NER factors are not required to remove this class of lesions) [41]. As shown in Figure 7D, *Ercc1^−/−^* cells are highly sensitive to MMC treatment. All the other NER mutant cells, including the *Ttda^−/−^* cells, are not sensitive. Since most oxidative DNA lesions are removed by the base excision repair (BER) genes [42], we wondered whether the oxidative DNA damage hypersensitivity could be due to reduced expression of BER genes, as a consequence of the low TFIIH level in *Ttda^−/−^* cells. To that aim we analyzed the expression of the core BER genes by RT-qPCR, since the absence of single oxidative damage-specific glycosylases does not cause cellular hyper-sensitivity due to (partial) redundant glycosylases [43]. As shown in Figure S5B, none of the BER genes were lower expressed in the *Ttda^−/−^* cells. To further investigate a possible general BER defect in Ttda^−/−^ cells, we tested for alkylating DNA damage sensitivity. Apart from the initial recognizing glycosylases, further processing of these lesions follows the same route as for oxidative DNA damage. To that aim we treated the cells with varying concentrations of Methyl methanesulfonate (MMS). In contrast to oxidative DNA damage, Ttda^−/−^ appeared not hypersensitive to this agent (Figure 7E). Together our data unambiguously establish a function for TTDA and likely for the entire TFIIH complex in the tolerance to oxidative DNA damage.

## Discussion

In an attempt to create a *Ttda* knock-out mouse model, we have shown that this gene is essential for embryonic development. Contrary to expectations, *Ttda^−/−^* mice die *in utero* between 10.5 days of gestation and birth. The lethality observed in *Ttda* ^−/−^ embryos obviously differs from the phenotype observed in TTD-A patients, whom have relatively mild TTD features. The fact that differential expression levels of neighboring genes did not correlate with lethality demonstrates that this lethality was not due to any inadvertent effect of the targeting strategy. Surprisingly, viable cells derived from *Ttda^−/−^* embryos are completely NER deficient, again in contrast to the mild NER defect observed in TTD-A patient cells. These findings indicate that TTDA's role in NER is in fact essential, and not auxiliary as previously suggested. Finally, *Ttda^−/−^* cells appeared hyper-sensitive to oxidative DNA damage, a finding not commonly associated with NER-deficiency. Both the requirement of Ttda for embryonic development and its function in oxidative DNA damage defense suggest that Ttda not only has a function in NER but also in several other processes, thereby challenging the NER-specific role of TTDA previously postulated.

### TTDA has a pivotal role in NER

Analysis of NER parameters in embryonic *Ttda^−/−^* cells revealed a remarkably severe NER-deficiency and includes extreme UV-hypersensitivity, absence of UV-induced UDS and defective in removing UV-induced lesion (comparable to *Xpa^−/−^* cells). This complete NER-deficient phenotype is in striking contrast to the only mild NER defect seen in human TTD-A cells. The NER-deficiency observed cannot be explained by a cell-type specific UV-response, since MEFs and ES cells are equally UV-sensitive. In a previous study we noted very low levels of all subunits of TFIIH in human TTD-A patient cells and suggested that this contributes to the associated partial NER defect [16]. MEFs derived from a previously generated TTD mouse model [26] — mimicking a known human TTD-causative point mutation (R722W) in the mouse *Xpd* locus — are only slightly sensitive to UV irradiation and exhibit a mild UDS defect and have decreased TFIIH levels [28]. Therefore, complete NER-deficiency appears to be specific to the *Ttda^−/−^* cells and not a general TTD-associated phenotype caused by a lower level of TFIIH. Since the NER-phenotype of *Xpd* TTD mice closely mimics the partial repair deficiency features in human XP-D TTD cells, it is thus unlikely that this discrepancy in the severity of the NER-phenotype between man and mice is a species-specific phenomenon of increased NER-deficiency in murine cells. Our dynamic *in vivo* studies have revealed that the TTDA defect is located at a stage prior to stable association of TFIIH with the NER initiation complex, containing XPC (Figure 2E). However, in contrast to NER, TTDA seems dispensable for loading TFIIH onto promoter sequences for transcription initiation of RNA pol I and II, in view of the fact that *Ttda^−/−^* cells are viable and do not show a proliferation defect.

### TTD-A patients express partially functional TTDA protein

Knock-down of the resident TTDA transcripts in TTD-A patient cells rendered these cells extremely UV-sensitive, with the same level of sensitivity as completely NER-deficient XP-A cells and similar to the *Ttda^−/−^* MEFs. From these data we conclude that in humans the mutant TTDA protein are partially functional. This hypothesis is further substantiated by the finding that the GFP-tagged TTDA^R56X^ protein (homozygous mutation in patient TTD99RO) was able to accumulate at sites of UV-induced damages, in line with the suggested presence of residual activity of mutant TTDA proteins in patient TTD-A cells.

Our data suggest that the TTDA protein in TTD13/14PV cells is partially functional. This observation is particularly intriguing, since it has been suggested that no TTDA protein is expressed in these cells, due to the homozygous translational start-site mutation [15]. However, the fact that depletion of the mutant TTDA mRNA by targeted shRNA interference further enhanced UV sensitivity, suggests that this mutant mRNA is still able to generate a partially functional TTDA protein. One way to explain this phenomenon is by assuming that despite this ATG mutation, some TTDA protein is still being produced by initiating from an in-frame downstream ATG (codon 16). The usage of such alternative start site may produce low levels of an N-terminally truncated TTDA protein, sufficient to rescue lethality and complete NER-deficiency. Indeed, over expression of a 16 amino-acid N-terminally truncated mutant *TTDA*, rescued the UDS defect of *Ttda^−/−^* cells (Figure 5D, 5E) and argues for partial functional production of N-terminally truncated TTDA in TTD12/14PV patient cells. Interestingly, it has been shown (both in yeast and humans) that the N-terminal domain of TTDA is important for binding to the TFIIH subunits XPD and p52 [17], [44] and for stimulating the ATPase-activity of the XPB subunit [17]. Furthermore, this interaction is also critical for the TFIIH stability. Apparently, the truncated Ttda protein is nevertheless able to carry out part of its function to permit residual NER.

### Ttda functions in oxidative DNA damage defense

Defects in multiple DNA repair systems may cause synergistic effects or even synthetic lethality [45]. For example, severe developmental and premature aging problems have been seen in KO mouse models of DNA repair factors that function in independent repair pathways, such as ERCC1 (which functions in NER and inter-strand cross-link repair) and *Xpa^−/−^ Csb^m/m^* double KOs (which is defective in GG-NER, and presumably also in the broad TCR pathway) [46], [47]. It has been suggested that endogenously produced DNA lesions (e.g. from reactive oxygen species (ROS) or lipid peroxidation byproducts) that cannot be removed because of the repair defect, are in part responsible for the phenotype observed. Here we have shown that *Ttda^−/−^* cells are sensitive to several oxidizing agents to the same extent as *Csb^−/−^* ES cells [30]. Based on these results, we suggest that *Ttda^−/−^* embryos are confronted with unrepaired endogenous oxidative lesions, possibly generated by low but continuous exposure to ROS during development. This compromised repair of oxidative DNA lesions may contribute to the observed lethal phenotype observed in the *Ttda^−/−^* embryos. Importantly, this reduced resistance to oxidative DNA damage is likely not caused by a general (core) BER defect. Previously, it was suggested that TFIIH is implicated in coordinating incision of lesion-stalled transcription complexes [48] and that some oxidative DNA lesions are processed by transcription-coupled repair [49]. It is thus possible that TTDA (TFIIH) is involved in a specific — thus far uncharacterized — transcription-coupled repair process of oxidative DNA damage.

### Lethal phenotype of *Ttda* knock-out mice

The fact that TTDA has a pivotal role not only in NER but also in oxidative DNA damage defense and transcription, argues for a function of TTDA in various DNA metabolizing processes, causing synergistic effects when inactivated. However, full NER-deficiency in combination with defects in oxidative DNA damage repair — as in *Xpa^−/−^ Csb^−/^* ^−^ double KO mice — does not lead to embryonic lethality. In this mouse model pups are born, but they progressively develop very severe neurologic symptoms and premature aging features. It is thus likely that not only the repair functions contribute to the lethal phenotype of *Ttda^−/−^* animals, but that also its function in transcription is involved. Nevertheless, human TTD-A patient derived cells also express low levels of TFIIH, but have only limited post-natal developmental problems [23]. It is possible that transcription is more demanding during mouse embryogenesis than in the human embryo, due to its more rapid development. In this respect it should be noted that TTD-causing mutations in the human *XPD* gene are associated with impaired placental development and other gestational complications [50].

It has been shown that TTDA is dispensable for mammalian *in vitro* transcription [17]. Nevertheless, TTDA was originally found to be present in the pre-initiation complex [20] and reconstituted transcription assays have demonstrated that TTDA stimulates this reaction [19]. It has been hypothesized that mutations affecting XPD's function in DNA repair cause the disorder XP — associated with a 1000-fold increased risk of skin cancer — while mutations affecting XPD's role in RNA Polymerase II-mediated transcription lead to TTD-specific features: brittle hair and nails, and scaly skin [51]. TTD features in TTD-A patients are relatively mild compared to those seen in XPD-associated TTD patients. The mild TTD-phenotype suggests that the role of TTDA in transcription is plausible, but also that it is not the only cause for embryonic lethality. We have demonstrated that *Ttda* KO cells have a low steady state level of TFIIH and accordingly have a lower transcriptional activity (Figure 6C and 6D). The low TFIIH quantity does not seem to be the sole cause of embryonic lethality, since similar low levels of TFIIH are observed in TTD-A patient cell lines, which are compatible with life. However, the notion of reduced transcriptional activity in *Ttda^−/−^* cells argues that this feature may contribute to embryonic lethality. For instance, during certain stages of embryonic development which requires high transcriptional capacity, normal embryogenesis may be compromised. Moreover, it cannot be excluded that mutations in *TTDA* affect the transcription of a subset of specific genes, as shown in cells with XPD-associated TTD mutations defective in activated-transcription of nuclear receptors [52]. In this scenario, the expression of specific genes, essential for development of the embryo might be disturbed hindering proper embryogenesis and finally inducing in utero death. In both cases, TTDA function appears to extend beyond the previously suggested main function in NER, as it is also important for both development and viability.

Our data clearly show that TTDA has an essential function in NER. The rather mild TTD-phenotype observed in TTD-A patients is due to the presence of partly functional mutant proteins. The sensitivity to endogenously produced oxidative DNA lesions in *Ttda^−/−^* cells suggests that TTDA (and likely the entire TFIIH) has additional functions in DNA repair extending beyond NER. The lethal phenotype observed in *Ttda^−/−^* embryos is possibly the result of several defects, such as insufficient levels of TFIIH needed for transcription in highly proliferative tissues, impairment in the activated transcription of specific genes, and unrepaired lesions — induced either by UV or endogenously by oxidizing agents which are relevant for cancer as well as aging.

## Materials and Methods

### Ethics statement

All animal work was conducted according to the Federation of European Laboratory Animal Science Associations (FELASA) ethical requirements and with respect of the 3R animal welfare rules.

### Construction of the *mTtda* targeting construct

The knock-out targeting vector (backbone Puc18) contained a 12.5 Kb XbaI fragment of mouse genomic DNA (isogenic to 129Ola) harboring the entire *Ttda* locus. The complete exon 3 (i.e. most of the protein coding sequence) was excised, using BalI digestion, and replaced with a neomycin gene-expression cassette, flanked by two LoxP sites [53] and used as a dominant selectable marker. The dominant marker was inserted in the same transcriptional orientation as the *Ttda* gene.

### Gene targeting

ES cells (129Ola, subclone IB10) were cultured in BRL-conditioned medium supplemented with 1,000 U/ml leukemia inhibitory factor (LIF). A total of 20 µg of the SalI linearized targeting vector was electroporated into approximately 10^7^ ES cells in 500 µl. Selection with 0.2 µg/ml G418 was started 24 h after electroporation. G418 resistant clones were isolated after 8–10 days. Screening for homologous recombinants was performed using DNA blot analyses of NheI digested DNA with a 1000 bp 5′ external probe A (see Figure 1A). 13 ES clones out of 130 G418-resistant clones, had a correctly targeted *Ttda* allele. Two of the 13 correctly targeted ES clones were checked for proper karyotype and injected into blastocysts from C57bl/6 mice and then transplanted into B10/CBA foster mothers. Chimeric mice were further crossed, and germ line transmission of the targeted allele to offspring was genotyped by PCR (Figure 1). Primer sequences are available on request.

### Cell culture and treatments

#### Cell culture

ES cells: IB10 (wild-type), *Xpb-YFP^+f/+f^*, *Xpb-YFP^+f/+f^ Ttda* **^+/−^**, *Xpb-YFP^+f/+f^ Ttda* **^−/−^**, *Ttda* **^+/−^**, *Ttda* **^−/−^**, *Xpa* **^−/−^**, *Csb^−/−^* and *Ercc1^−/−^*. They were cultured in a 1∶1 mixture of DMEM (Lonza) and BRL conditioned medium with 10% foetal calf serum (FCS), 1% penicillin-streptomycin (pen-strep; Gibco), 1% non-essential amino acids, 0.1% β-mercaptoethanol (Invitrogen) and 0.01% leukemia inhibitor factor (home-made) at 37°C, 20% O_2_ and 5% CO_2_. The dishes were pre-coated with a 0.1% gelatine solution in water.

MEFs: wild-type, *Xpb-YFP^+f/+f^*, *Xpb-YFP^+f/+f^ Ttda* **^+/−^**, *Xpb-YFP^+f/+f^*, *Ttda* **^−/−^**, *Ttda* **^+/−^**, *Ttda* **^−/^** ^−^, *Ttda* **^−/−^** expressing TTDA^WT^-GFP; TTDA^L21P^-GFP or TTDA^R56X^-GFP, *Xpa* **^−/−^**, *Csb^−/−^* and *Ercc1^−/−^*, were cultured in a 1∶1 mixture of DMEM and Ham's F10 (Lonza) with 10% FCS and 1% pen-strep at 37°C, 20% O_2_ and 5% CO_2_.

SV40-immortalized human fibroblasts: TTD1BR-sv (TTD-A), TTD1BR-sv stably expressing shRNA (#non-targeting (NT), #3398, #3399, #3400, #3401 and #3402) and TTD1BR-sv stably expressing either TTDA^WT^-GFP or TTDA^A56X^-GFP,were cultured in a 1∶1 mixture of DMEM and Ham's F10 with 10% FCS and 1% pen-strep at 37°C, 20% O_2_ and 5% CO_2_.

#### Treatments

For the local UV irradiation, cells were treated with a UV-C germicidal lamp (254 nm; Phillips) through a 5-µm microporous filter at 60 J/m^2^.

For transiently expression of DNA construct in MEFs, transfections were performed using jetPEITM (Polyplus transfections) according to the manufacturer's protocols.

To determine the cell cycle stage, cells were incubated for 1 hour (prior to immuno fluorescence) with culture medium containing 0.1 µM 5-ethynyl-2′-deoxyuridine (EdU; Invitrogen). After labelling with EdU, an UDS experiment (fluorescently labelled) was performed followed by an immuno fluorescence experiment (described below).

### Embryo isolation

#### 3.5-day-old embryos to generate ES cells

Blastocysts were isolated 3.5 days after fertilization and grown in a 24 wells plate using irradiated MEFs (20 Gy) as a feeder layer in Knock-out ES medium (Invitrogen) with 20% knock-out serum replacement (Invitrogen), 100 U/ml penicillin/streptomycin, non-essential amino acids (Invitrogen) 0.1 mM β-mercaptoethanol (Invitrogen), 5000 U/ml Leukaemia inhibitory factor (LIF) and 50 µM MEK1 inhibitor (Cell Signaling Technology). Colonies originated from blastocysts were trypsinized and cultured further on gelatine pre-coated culture dishes at 37°C, 20% O_2_ and 5% CO_2_.

#### 10.5-day-old embryos to generate MEFs

Embryos were isolated 10.5 days after fertilization, sheared using a pipette to detach the different cells and cultured in MEF medium at 37°C, 5% O_2_ and 5% CO_2_. Cells were cultured further under high oxygen conditions (20%) until an established cell line was formed.

### RNA isolation and real-time quantitative PCR

Total RNA was purified from ES cells and 11.5-day-old embryos using the RNeasy Mini Kit (Qiagen). cDNA was created from 2 µg of RNA using an RT kit (Invitrogen) and random primers (Invitrogen). 5 µl of this cDNA was used in the following reaction with 29 nM sense and antisense primer (primer sequences available on request). The cDNA was amplified by real-time quantitative PCR (RT-qPCR) in 25 µl reactions using platinum Taq polymerase and SYBR green according to manufacturer's protocol (Invitrogen) with the c1000 Thermal Cycler (Bio-Rad). Reaction conditions were: 95°C for 3 min, followed by 40 cycles of 95°C for 15 sec, 60°C for 30 s and 72°C for 30 sec and ending with 95°C for 1 min. Expression levels were normalized to *Gapdh* using the method described by Pfaffl [54]. Specificity of the reactions was confirmed by analysis of the RT-qPCR melt curves.

### Cell survival

#### Colony survival

Sensitivity of ES cells, MEFs and human cells to increasing doses of DNA damaging agents was determined as described previously [55]. Briefly, cells were plated in 6 cm dishes, in quadruplicate (untreated) or triplicate (treated). After 12–16 hours, cells were irradiated with a single dose ranging between 0 and 8 Gy using a ^137^Cs source or UV irradiation (0–8 J/m^2^; 254 nm; Philips TUV lamp) or treated for 1 hour with MMC (0–0.6 µg/ml; Kyowa), or treated for 1 hour with MMS (0–2 mM; Sigma) or KBrO_3_ (0–0.8 µg/ml; Sigma). After approximately 7 days, the colonies were fixed and stained with 0.1% Brilliant Blue R (Sigma) and were counted. The survival was plotted as the percentage of colonies obtained after treatment compared to the mean number of colonies from the non-treated samples (set at 100%).

#### [methyl-^3^H]-thymidine survival

MEFs were plated in 6-well culture dishes (1×10^4^ cells per well) in quadruplicate (0 J/m^2^) or triplicate (others) in 3 ml medium. Two days after seeding, cells were washed with PBS and UV irradiated (0–8 J/m^2^). Five days after irradiation cells were pulse-labeled with [*methyl*-^3^H]-thymidine (40–60 Ci/mmol; 5 µCi/ml; Amersham Biosciences), chased for 30 minutes in unlabeled medium, washed with PBS, lyzed in 0.25 M NaOH and harvested. Cell lysates were transferred into scintillation flasks and supplemented with 7.5 ml Hionic Fluor scintillation fluid (Packard). Each sample was counted in the scintillation counter for 10 minutes and results were expressed as the percentage of counts obtained from the non-treated dishes (set as 100%).

### UV-induced UDS and RRS

#### Autoradiography

For the UDS and RRS assay [56] 1×10^6^ cells were seeded onto 24 mm cover slips and after 2 days UV irradiated with 16 J/m^2^. For UDS; cells were incubated (directly after UV irradiation) for 2 hours in culture medium containing 10 µCi/ml [*methyl*-^3^H] Thymidine (110 Ci/mmol; Amersham Biosciences), washed with PBS and fixed. For RRS, 16 hours after UV irradiation cells were incubated for 2 hours in culture medium containing 10 µCi/ml [^3^H]Uridine (110 Ci/mmol; Amersham Biosciences), washed with PBS and fixed. In both assays the cover slips with radioactively labeled cells were mounted onto slides and dipped in a photosensitive emulsion (Ilford K2). After 2 to 7 days exposure, slides were developed and stained. Repair capacity was quantified by the number of auto- radiographic grains above the nuclei of at least 25 cells. UDS or RRS levels were expressed as the percentage of the number of grains above wild-type cells (set at 100%), assayed in parallel.

#### Fluorescent assay

For UDS 1×10^6^ cells were seeded onto 24 mm cover slips and after 2 days UV irradiated with 16 J/m^2^. The cells were washed once with PBS and incubated for 2 h in culture medium containing 0.1 µM 5-ethynyl-2′-deoxyuridine (EdU; Invitrogen). After EdU incorporation, cells were washed twice with PBS followed by fixation in 1 ml of 4% formaldehyde in PBS. Cells were washed twice with 3% BSA in PBS and permeabilized for 20 minutes in 0.5% Triton in PBS and washed once with PBS. Cells were incubated for 30 minutes with fluorescent dye coupling buffer containing 10 mM CuSO4 and Alexa Fluor 594 azide (Qlick-iTTM, Invitrogen). After washing with PBS, cells were mounted in vectashield. UDS levels were expressed as the average fluorescence intensity in the nucleus of wild-type cells, which was set at 100%. The mean fluorescence is determined from at least 25 cells.

### Transcription measured by EU incorporation

ES cells and MEFs were grown in a 6 cm dish and cultured for 2 days prior to the experiments. The cells were washed once with PBS and incubated for 30 minutes or 2 hours in culture medium containing 0.1 µM 5-ethynyl-uridine (EU). After EU incorporation, cells were treated in the same way as described above (UDS; fluorescent assay).

### Quantification of 6-4 PP and CPD UV-photoproducts by ELISA

70–80% confluent cultures of MEFs to be analyzed were washed with PBS, UV irradiated (8 J/m^2^) and incubated for various time points (1 to 16 hours). Cells were harvested in PBS and DNA was isolated using QIAamp DNA Blood Mini Kit (QIAGEN). DNA concentrations were determined by measuring the optical density at 260 nm. 96-well polyvinylchloride flat-bottom micro titer plates were precoated with 0.003% Protamine Sulfate, 50 µl/well and dried in the dark overnight at 37°C (Sigma). DNA samples were denatured for 10 minutes at 95°C and immediately cooled on ice for 20 min. 50 µl/well of vortexed DNA solution in H_2_O was loaded in the precoated 96-well plate to a final concentration of 6 µg/ml for the detection of 6-4PP. The plate was dried overnight at 37°C, then washed 5 times with PBS+0.05% Tween-20 (150 µl/well). The wells were pre-absorbed with PBS+2% FCS for 30 minutes at 37°C and subsequently washed 5 times with PBS/Tween-20 prior to incubation for 30 min at 37°C with 100 µl/well of primary antibody: 6-4PP (1∶1000; Bioconnect/MBL) diluted in PBS. After 5 washes with PBS/Tween-20, samples were incubated for 30 minutes at 37°C with 100 µl/well of secondary antibody: goat anti-mouse IgG (H+L) conjugated to HRP (1∶2000; Southern Biotech). After 5 washes with PBS/Tween-20, samples were treated with 100 µl/well Citrate-phosphate buffer (24 mM C_6_H_8_O_7_.H_2_O and 41 mM Na_2_HPO_4_.2H_2_O; Sigma). Samples were then incubated with 100 µl/well of freshly made ODP buffer (0.4% o-Phenylene damine and 0.02% citrate-phosphate buffer; Sigma) at 37°C for 30 min. The reaction was stopped by adding 50 µl/well of 2 M H_2_SO_4_ and absorbance was immediately measured at 490 nm.

### Confocal microscopy

#### Multiphoton laser-induced DNA damage

Laser-induced DNA damage on MEFs expressing the Xpb-YFP fusion protein was performed using a Chameleon Ultra II modelocked Ti:Sapphire laser system (Coherent Inc) that was directly coupled to an LSM 510 NLO microscope (Zeiss) to obtain an 800-nm, pulsed (80 MHz) output of 26 mW. Single nuclei targeted with the multiphoton laser were exposed for 56.6 ms (via 25 iterations) over a 2.6 micrometer diameter disc.

#### UV laser-induced DNA damage

For UV laser irradiation a 2 mW pulsed (7.8 kHz) diode pumped solid state laser emitting at 266 nm (Rapp OptoElectronic, Hamburg GmbH) was connected to a Leica SP5 confocal microscope. For local UV-C irradiation experiments cells were grown on 25 mm diameter quartz coverslips (010191T-AB, SPI supplies) and images were obtained using a 100× quartz objective. UV-C laser irradiation was performed as previously described [31].

#### Analysis

The accumulation of tagged proteins at locally induced damaged regions was obtained from a series of time-lapse images by measuring the average fluorescence within a circular region matching the laser-damaged area (F^LD^). The average fluorescence of the nucleus and the background fluorescence levels were also measured. To calculate the fluorescence signal due to the accumulation of tagged proteins within the damaged area, the average fluorescence estimated outside of the damaged region (F^Nucleus^)(but still within the nucleus) was subtracted from F^LD^. The obtained fluorecence: F^LD true^ is derived from: F^LD true^ = F^LD^ - F^Nucleus – LD^. Dividing F^LD true^ by the total nuclear fluorescence corrected for background, resulted in the normalized fluorescence signal at the local damage area: F^Norm.LD true^ = F^LD true^/F^N^.

### Immunofluorescence

Cells were grown on glass cover slips (24 mm) for three days prior to the experiments and fixed with 2% paraformaldehyde (Sigma) at 37°C for 15 minutes. Cover slips were washed three times for 5 minutes with PBS containing 0.1% Triton X-100 (Sigma). To visualize the DNA photoproducts, nuclear DNA was denatured by incubation with 0.07 N NaOH (Sigma) at room temperature for 5 minutes and washed tree times for 5 minutes with 0.1% Triton X-100 and subsequently for 5 minutes with PBS^+^ (PBS containing 0.15% glycine (Sigma) and 0.5% BSA (Sigma)). Cells were incubated at room temperature with primary antibodies for 2 hrs in a moist chamber. Subsequently, cover slips were washed three times with PBS/Triton X-100 and PBS^+^, incubated 1 hour with secondary antibodies at room temperature and again washed three times in PBS/Triton X-100. Samples were embedded in Vectashield mounting medium (Vector Laboratories). Images were obtained using a confocal laser scanning microscope (LSM 510; Zeiss). The primary antibodies used for this procedure were mouse anti-CPD (1∶1000; TDM-2; BioConnect/MBL), rabbit anti-GFP (1∶1000; ab290; Abcam), rabbit anti XPB (1∶1000; S19; Santa Cruz), rabbit anti XPC (1∶200; fraction 5; home-made), mouse anti γH2AX (1∶1000; 07-164; Millipore) and mouse anti-MDC1 (1∶1000; MDC1-50; Abcam). The secondary antibodies used were Alexa Fluor 594 goat anti-mouse IgG (H+L) (1∶1000; Molecular probes), Alexa Fluor 594 goat anti-rabbit IgG (H+L) (1∶1000; Molecular probes), Alexa Fluor 488 goat anti-rabbit IgG (H+L) (1∶1000; Molecular probes), Alexa Fluor 488 goat anti-mouse IgG (H+L) (1∶1000; Molecular probes) and Alexa Fluor 647 azide (1∶400; invitrogen).

### Proliferation assay

To measure proliferation in MEFs, equal number of cells from early passages (p2) were plated in 6 cm culture dishes (approximately 1×10^4^ cells per well) in triplicate in 3 ml medium (day 0). The medium was changed every 2 days cells and cells were counted 1, 3, 5 and 6 days after seeding, using a cell counter (Beckman Coulter Z2).

## Supporting Information

Figure S1 Accumulation of XpbYFP and Xpc at locally induced DNA damaged regions. Accumulation kinetics of XpbYFP to local Multi Photon (MP) damage in a wild-type, *Ttda^+/−^* and *Ttda^−/−^* backgrounds. Graphs represents the mean YFP-derived fluorescence intensity at the damaged spot at the indicated time points from 12 cells.
(TIF)

Figure S2 Quantitative immune-fluorescence to determine the relative amount of TFIIH. Representative confocal microscope pictures of MRC5-sv (wild-type), TTD1BR-sv (TTD-A) and TTD1BR-sv cells stably expressing shRNA (#3398 or #3402). Cells were stained with DAPI (left), anti-XPB (TFIIH subunit) (middle) and anti-MDC1 (internal control) (right).
(TIF)

Figure S3 Mutant TTDA-GFP accumulation at locally UV-induced regions. (A) Colony forming ability after different doses of UV of wild-type, *Ttda^+/−^*. *Ttda^−/−^*, *Ttda^−/−^* stably transfected with TTDA^WT^-GFP and *Xpa^−/−^* ES cells. The percentage of surviving cells was plotted against the applied UV-dose, measured by counting surviving colonies of two independent experiments. The error bars indicate the SEM. (B) Immuno-fluorescent analysis on TTD1BR-sv cells stably expressing either TTDA^WT^-GFP or TTDA^R56X^-GFP. Cells were seeded on cover slips and the next day irradiated locally with 60 J/m^2^ through a filter containing 5 µm pores. Cells were fixed 1 hour after UV and immuno-fluorescent staining was performed using antibodies against CPDs (damage marker, red) and GFP (green).
(TIF)

Figure S4 Gene expression levels of *Ttda^−/−^* 11.5-day-old embryos. Relative expression levels of mRNAs neighboring genes encoding Synaptojamin 2 (*Synj2*), Serine active site containing 1 (*Serac1*) and Tubby like protein 4 (*Tulp4*) in *Ttda^−/−^* (n = 8), *Ttda^+/−^* (n = 8) and wild-type (n = 8) in embryos as determined by quantitative RT-PCR. The levels were normalized to *Gapdh* and the error bars indicate SEM between experiments.
(TIF)

Figure S5 Proliferation assay and gene expression levels of BER genes. (A) Equal number of cells (1×10^4^) were plated on 6 cm culture dishes in triplicate (day 0). The total number of cells was counted in wild-type (n = 2), *Ttda^+/−^* (n = 2) and *Ttda^−/−^* (n = 2) MEFs at different days after seeding. The error bars indicate the SEM. (B) Relative expression levels of mRNAs encoding Apurinic-apyrimidinic endonuclease 1 (*Apex1*), Flap structure-specific endonuclease 1 (*Fen1*), Ligase III (*Lig3*), Poly [ADP-ribose] polymerase 1 (*PARP1*), DNA polymerase beta (*PolB*) and X-ray repair cross-complementing protein 1 (*Xrcc1*) in wild-type (n = 8) and *Ttda^−/−^* (n = 8) 11.5-days-old embryos as determined by quantitative RT-PCR. The levels were normalized to *Gapdh* and the error bars indicate SEM between experiments.
(TIF)

Table S1 Genotyping of the offspring from matings of *Ttda^+/−LNL^* mice. Genotyping of offspring from matings of *Ttda^+/−LNL^* mice, distributed over male and females, obtained number and percentage of offspring compared to the theoretical expected figures assuming a Mendalian inheritence pattern.
(DOCX)
