# Supplementary figures and images for: Disruption of TTDA Results in Complete Nucleotide Excision Repair Deficiency and Embryonic Lethality
Source: PLoS Genet. 2013 Apr 18;9(4):e1003431. doi: 10.1371/journal.pgen.1003431 (PMC3630102; doi:10.1371/journal.pgen.1003431)

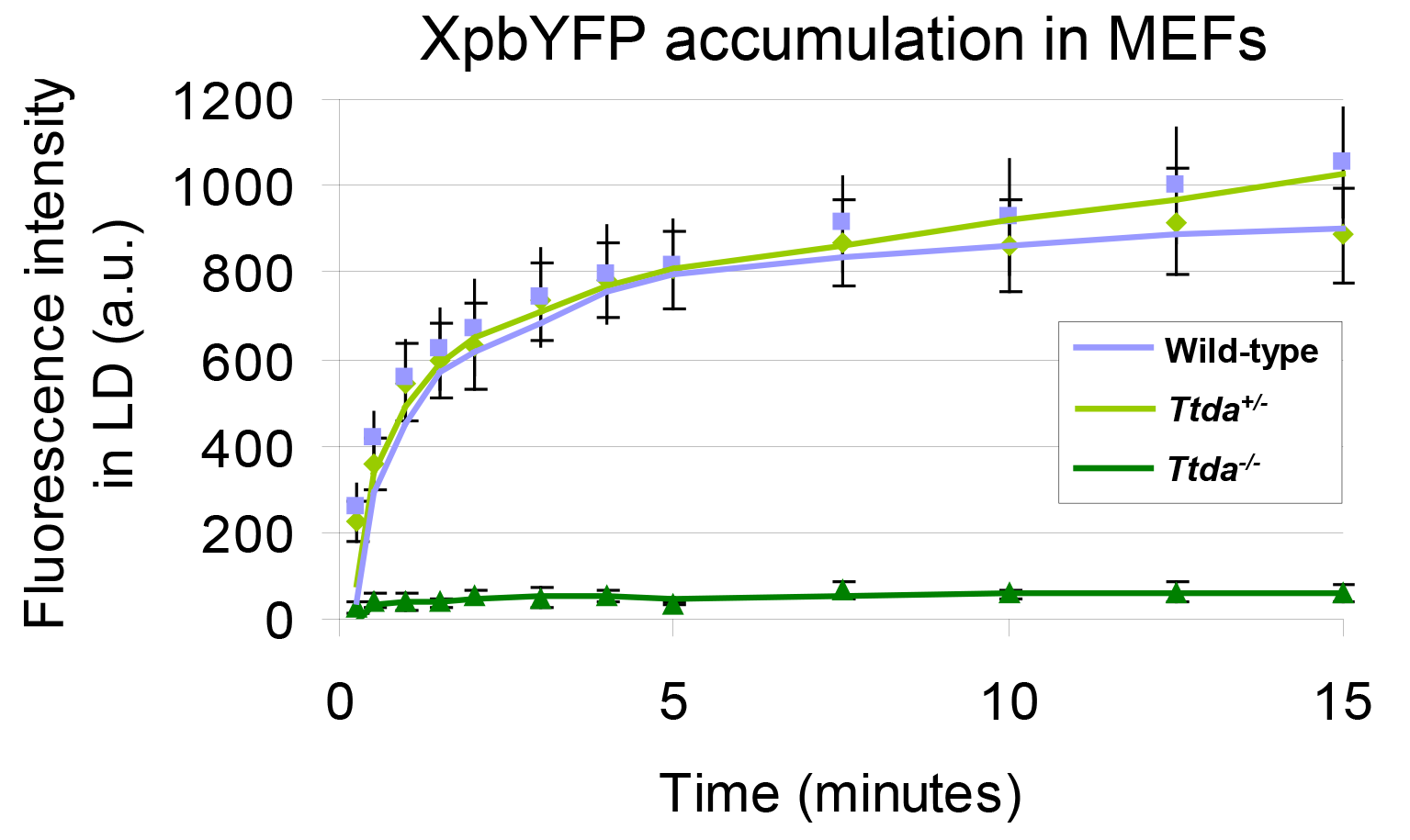

Supplement: Figure S1 — Accumulation of XpbYFP and Xpc at locally induced DNA damaged regions. Accumulation kinetics of XpbYFP to local Multi Photon (MP) damage in a wild-type, Ttda+/− and Ttda−/− backgrounds. Graphs represents the mean YFP-derived fluorescence intensity at the damaged spot at the indicated time points from 12 cells. (TIF) [file pgen.1003431.s001.tif]

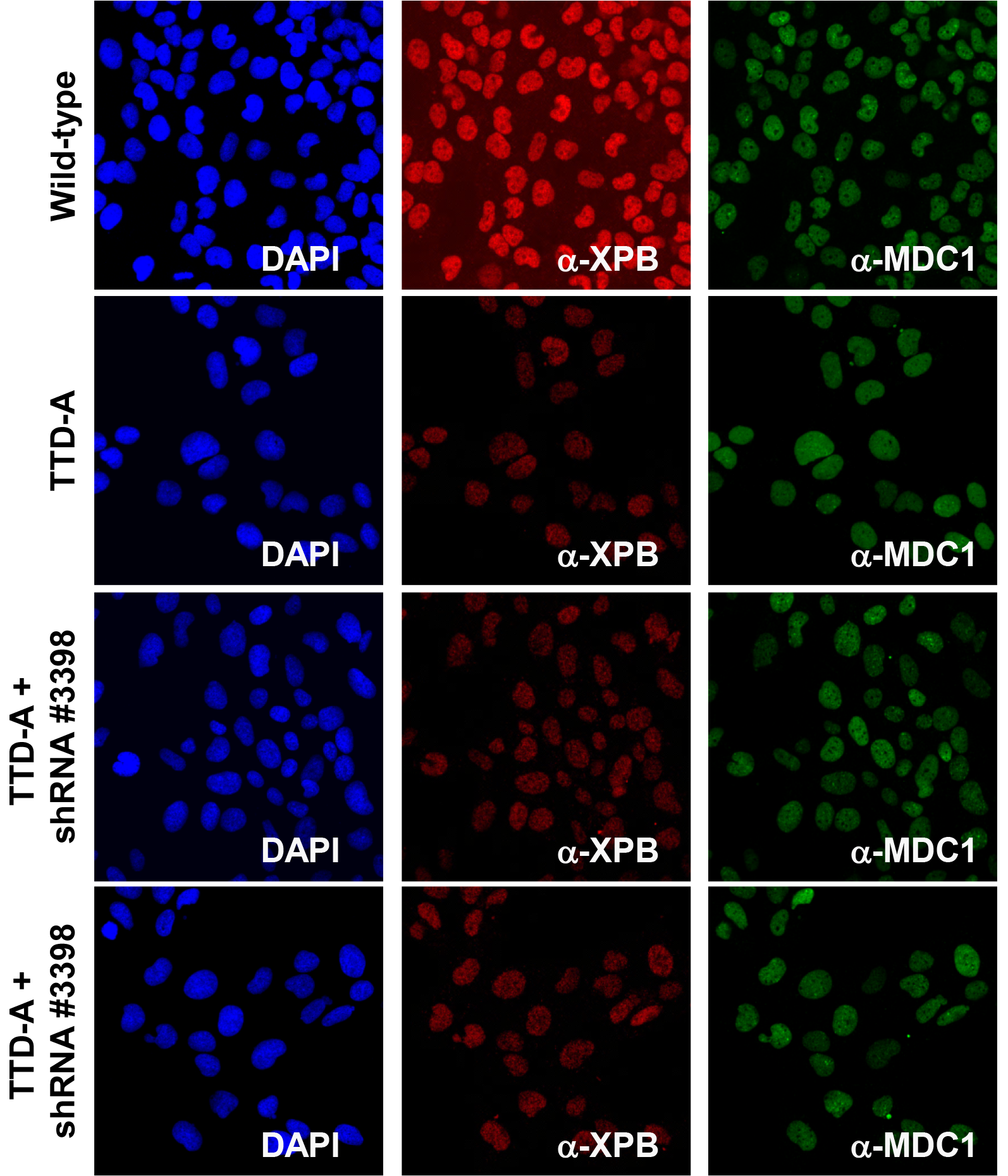

Supplement: Figure S2 — Quantitative immune-fluorescence to determine the relative amount of TFIIH. Representative confocal microscope pictures of MRC5-sv (wild-type), TTD1BR-sv (TTD-A) and TTD1BR-sv cells stably expressing shRNA (#3398 or #3402). Cells were stained with DAPI (left), anti-XPB (TFIIH subunit) (middle) and anti-MDC1 (internal control) (right). (TIF) [file pgen.1003431.s002.tif]

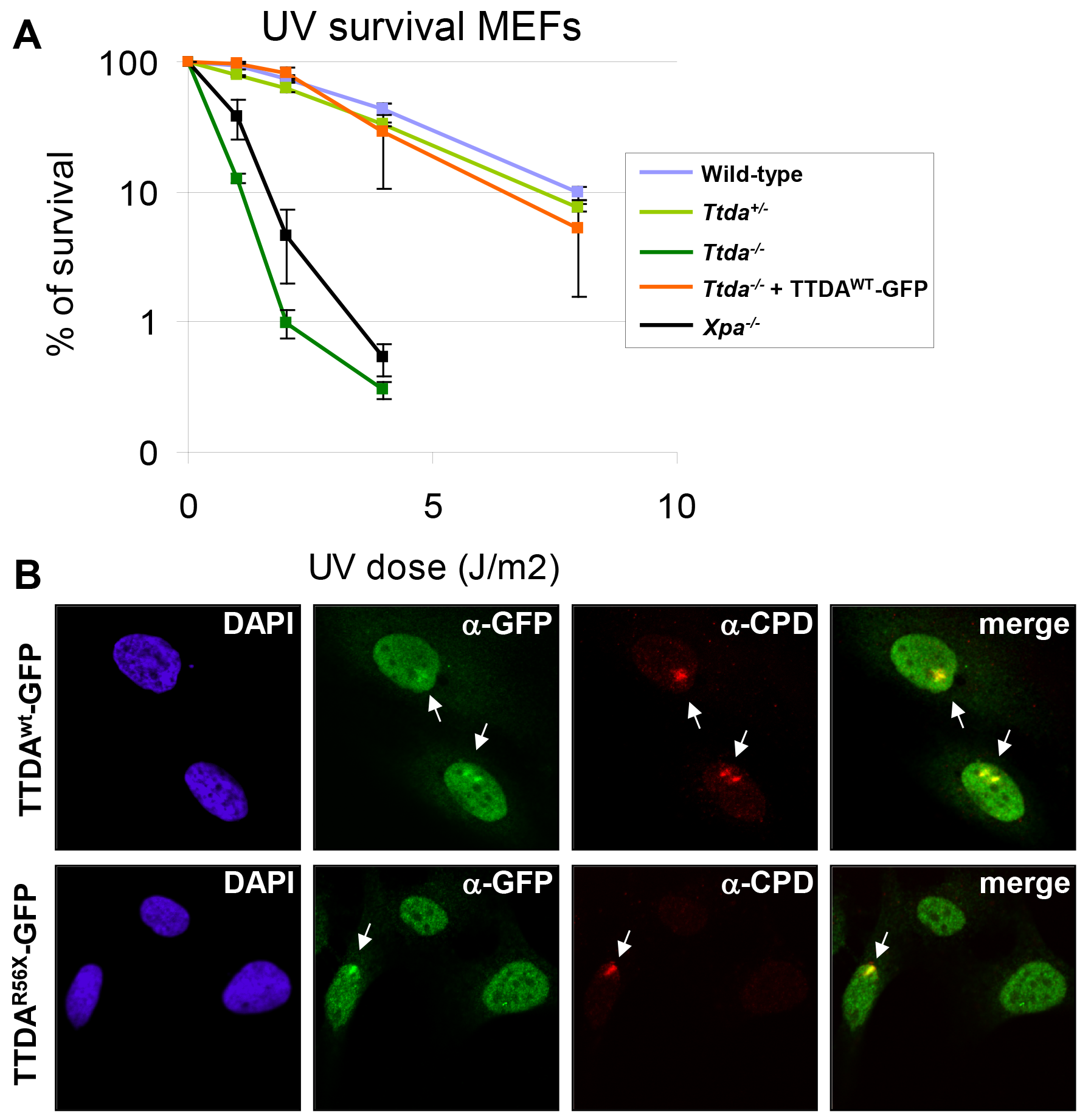

Supplement: Figure S3 — Mutant TTDA-GFP accumulation at locally UV-induced regions. (A) Colony forming ability after different doses of UV of wild-type, Ttda+/−. Ttda−/−, Ttda−/− stably transfected with TTDAWT-GFP and Xpa−/− ES cells. The percentage of surviving cells was plotted against the applied UV-dose, measured by counting surviving colonies of two independent experiments. The error bars indicate the SEM. (B) Immuno-fluorescent analysis on TTD1BR-sv cells stably expressing either TTDAWT-GFP or TTDAR56X-GFP. Cells were seeded on cover slips and the next day irradiated locally with 60 J/m2 through a filter containing 5 µm pores. Cells were fixed 1 hour after UV and immuno-fluorescent staining was performed using antibodies against CPDs (damage marker, red) and GFP (green). (TIF) [file pgen.1003431.s003.tif]

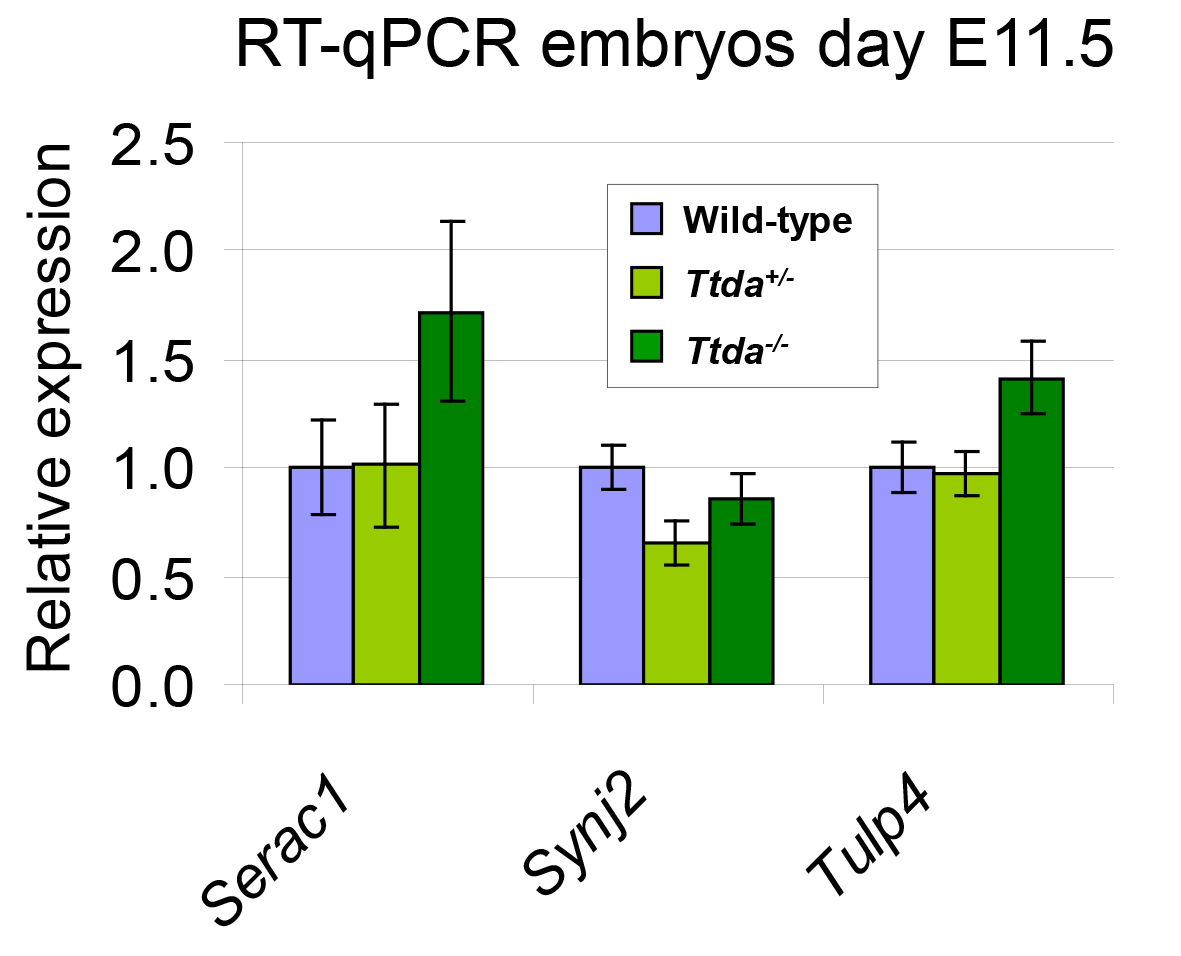

Supplement: Figure S4 — Gene expression levels of Ttda−/− 11.5-day-old embryos. Relative expression levels of mRNAs neighboring genes encoding Synaptojamin 2 (Synj2), Serine active site containing 1 (Serac1) and Tubby like protein 4 (Tulp4) in Ttda−/− (n = 8), Ttda+/− (n = 8) and wild-type (n = 8) in embryos as determined by quantitative RT-PCR. The levels were normalized to Gapdh and the error bars indicate SEM between experiments. (TIF) [file pgen.1003431.s004.tif]

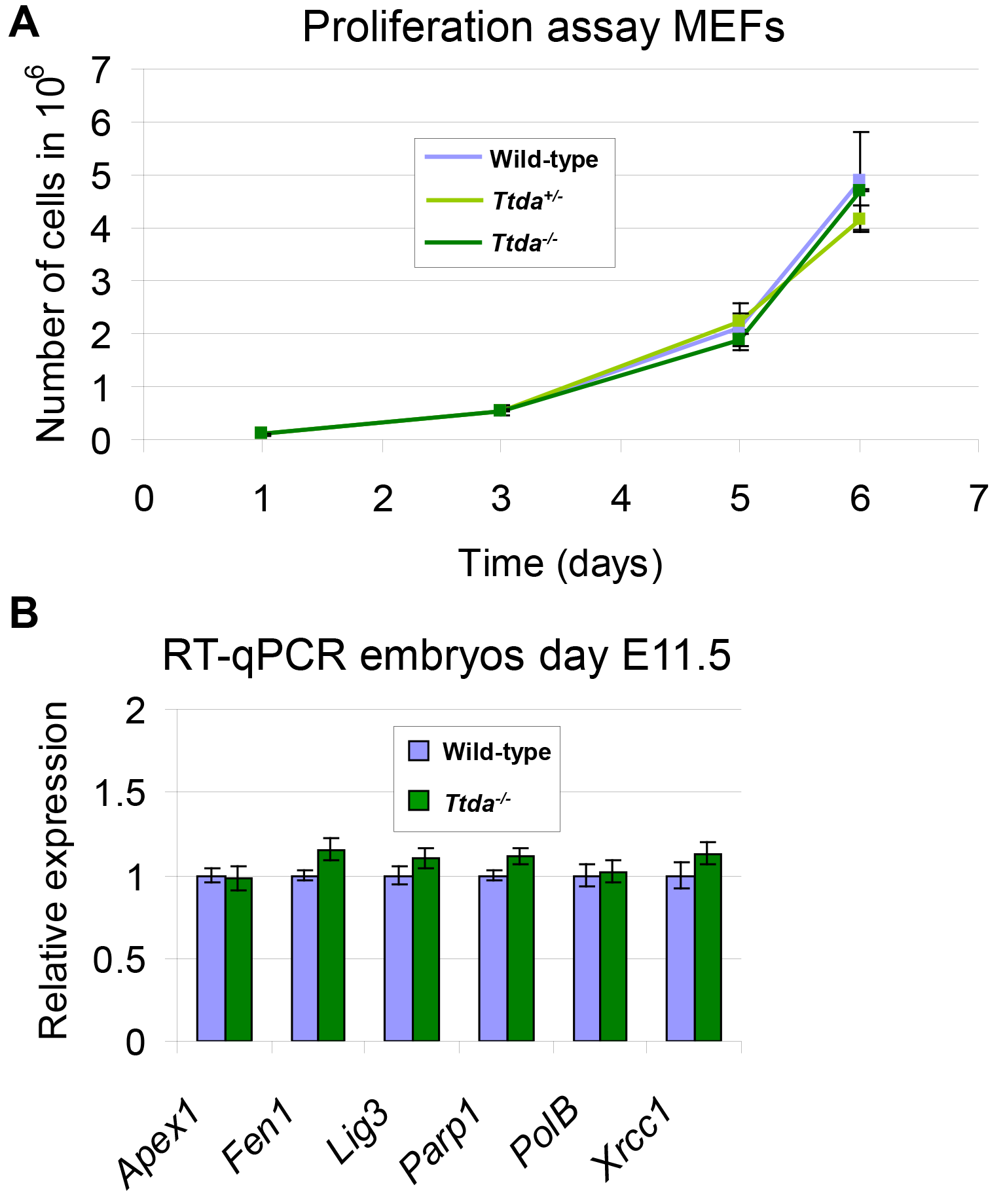

Supplement: Figure S5 — Proliferation assay and gene expression levels of BER genes. (A) Equal number of cells (1×10^4^) were plated on 6 cm culture dishes in triplicate (day 0). The total number of cells was counted in wild-type (n = 2), Ttda+/− (n = 2) and Ttda−/− (n = 2) MEFs at different days after seeding. The error bars indicate the SEM. (B) Relative expression levels of mRNAs encoding Apurinic-apyrimidinic endonuclease 1 (Apex1), Flap structure-specific endonuclease 1 (Fen1), Ligase III (Lig3), Poly [ADP-ribose] polymerase 1 (PARP1), DNA polymerase beta (PolB) and X-ray repair cross-complementing protein 1 (Xrcc1) in wild-type (n = 8) and Ttda−/− (n = 8) 11.5-days-old embryos as determined by quantitative RT-PCR. The levels were normalized to Gapdh and the error bars indicate SEM between experiments. (TIF) [file pgen.1003431.s005.tif]
