# Supplementary material for: Disruption of TTDA Results in Complete Nucleotide Excision Repair Deficiency and Embryonic Lethality
Source: PLoS Genet. 2013 Apr 18;9(4):e1003431. doi: 10.1371/journal.pgen.1003431 (PMC3630102; doi:10.1371/journal.pgen.1003431)
Supplement: Table S1 — Genotyping of the offspring from matings of Ttda+/−LNL mice. Genotyping of offspring from matings of Ttda+/−LNL mice, distributed over male and females, obtained number and percentage of offspring compared to the theoretical expected figures assuming a Mendalian inheritence pattern. (DOCX) [file pgen.1003431.s006.docx]

**Table S1. Genotyping of the offspring from matings of *Ttda^+/-LNL^* mice.**

|  | Female | Male | Total expected | Total found | Expected % of total | Found % of total |
| --- | --- | --- | --- | --- | --- | --- |
| Wild-type | 12 | 19 | 22 | 31 | 25% | 35% |
| *Ttda^+/-LNL^* | 26 | 32 | 45 | 58 | 50% | 65% |
| *Ttda^-LNL/-LNL^* | 0 | 0 | 22 | 0 | 25% | 0% |
| Total | 38 | 51 | 89 | 89 | 100% | 100% |
